# Fecal host biomarkers predicting severity of *Clostridioides difficile* infection

**DOI:** 10.1172/jci.insight.142976

**Published:** 2021-01-11

**Authors:** Makan Golizeh, Kaitlin Winter, Lucie Roussel, Marija Landekic, Mélanie Langelier, Vivian G. Loo, Momar Ndao, Donald C. Vinh

**Affiliations:** 1 Infectious Diseases and Immunity in Global Health Program, Research Institute of the McGill University Health Centre (RI-MUHC), Montréal, Québec, Canada.; 2 Department of Microbiology & Immunology and; 3 Department of Medicine, Faculty of Medicine and Health Sciences, McGill University, Montréal, Quebéc, Canada.; 4 Host-directed Immunotherapy to Fight Infectious disease (HI-FI) Program, Montréal, Québec, Canada.; 5 Division of Medical Microbiology, Department of Laboratory Medicine, MUHC, Montréal, Québec, Canada.

**Keywords:** Infectious disease, Inflammation, Bacterial infections, Proteomics

## Abstract

*Clostridioides difficile* is a major cause of health care–associated diarrhea. Severity ranges from mild to life-threatening, but this variability remains poorly understood. Microbiologic diagnosis of *C*. *difficile* infection (CDI) is straightforward but offers little insight into the patient’s prognosis or into pathophysiologic determinants of clinical trajectory. The aim of this study was to discover host-derived, CDI-specific fecal biomarkers involved in disease severity. Subjects without and with CDI diarrhea were recruited. CDI severity was based on Infectious Diseases Society of America/Society for Healthcare Epidemiology of America criteria. We developed a liquid chromatography tandem mass spectrometry approach to identify host-derived protein biomarkers from stool and applied it to diagnostic samples for cohort-wise comparison (CDI-negative vs. nonsevere CDI vs. severe CDI). Selected biomarkers were orthogonally confirmed and subsequently verified in a CDI mouse model. We identified a protein signature from stool, consisting of alpha-2-macroglobulin (A2MG), matrix metalloproteinase-7 (MMP-7), and alpha-1-antitrypsin (A1AT), that not only discriminates CDI-positive samples from non-CDI ones but also is potentially associated with disease severity. In the mouse model, this signature with the murine homologs of the corresponding proteins was also identified. A2MG, MMP-7, and A1AT serve as biomarkers in patients with CDI and define novel components of the host response that may determine disease severity.

## Graphical abstract

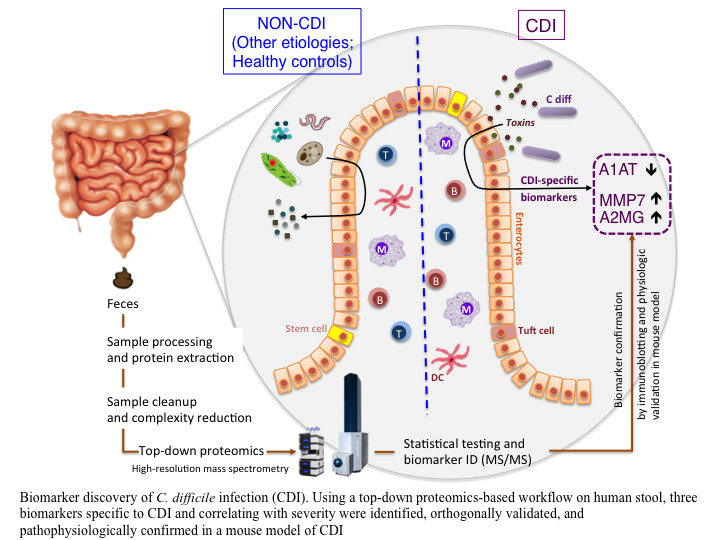

## Introduction

*Clostridioides difficile*, an anaerobic, Gram-positive, spore-forming bacterium transmitted by the fecal-oral route, is the leading cause of health care–associated diarrhea (incidence: 4.3–7.9/10,000 patient days) (1). In some regions, community rates are also high (approximately 12 cases/100,000 person years), and cases are increasingly identified from patients classically considered at low risk for disease, such as young adults without recent antibiotic exposures (2). Once *C*. *difficile* infection (CDI) develops, it is associated with increased length of hospital stay (approximately 3.6 days/episode) (1), costs (point estimates of $272 million in medical costs, $10 million in lost productivity) (2), morbidity (e.g., discharge to long-term care facility), and mortality (attributable mortality rate of 5%–10%) (1, 3). Thus, CDI is a significant health care burden.

### C.

*difficile* causes a spectrum of manifestations, ranging from asymptomatic colonization, to mild diarrhea (which may be self-limiting), to severe disease. The grade of severity can be assessed by various systems, including the Society for Healthcare Epidemiology of America (SHEA) and the Infectious Diseases Society of America (IDSA) criteria (3), in which severe disease is marked by hypotension, shock, ileus, megacolon, colectomy, admission to the intensive care unit, or death. The basis for this variability in clinical course remains incompletely understood. Epidemiologic studies have identified risk factors for CDI, including use and duration of antibiotics, increasing age (4), and compromised immune status (1, 3). Unfortunately, these factors are not sufficiently granular to inform on an individual’s prognosis, nor are they therapeutically actionable once disease is established. Microbiologic studies on *C*. *difficile* have identified bacterial strains (e.g., NAP1) and virulence factors that enable or enhance pathogenicity, which have contributed to the global trend of increased disease severity (5). However, strain type may account for regional/epidemic differences in severity, but it does not seem to account for the interindividual variability within a region affected by a dominant strain (4). Perturbation in the intestinal microbiome (dysbiosis) may also contribute to pathogenicity (6). However, interindividual variability in clinical evolution argues that there are host-related factors that are determinants of CDI outcome. A better biological understanding of these determinants is thus critical: the ability to pair microbial detection (diagnosis) with accurate identification of the patient’s clinical trajectory (prognosis) would provide guidance on clinical management. Moreover, identification of such biomarkers may provide insight into pathogenesis and determinants of disease outcome that are potentially tractable.

The identification of biomarkers to define clinical subsets of CDI has been previously pursued (4, 5, 7, 8). To date, most studies have been in a limited and targeted fashion, focusing on blood-borne biomarkers. Although some of these biomarkers, such as white blood cell count, C-reactive protein, and serum creatinine, are associated with poorer outcomes (9), they reflect the systemic nature of the ongoing disease process rather than being predictive of it. Further, these markers are not specific to CDI, nor do they give insight into the immunopathogenesis of disease.

Because CDI is the consequence of a microbe-host interaction at the level of the intestine, an analysis at this interface may be more informative, which can be accomplished via biomarker analysis of stool. Few studies with this approach exist, and they have primarily explored preselected fecal cytokines and/or limited proteins associated with inflammatory responses (e.g., lactoferrin, calprotectin) (10–17). These studies suggest a strong relationship between intestinal inflammatory host response and disease; they also establish proof of concept that analysis of proteins in fecal samples is feasible. However, because they are biased toward preselected targets, they risk missing biomarkers that are novel and/or CDI specific. Further, their correlation to clinical severity and outcome remains to be established.

In this study, we used an unbiased proteomics approach using liquid chromatography tandem mass spectrometry (LC-MS/MS) to determine human protein biomarkers from diagnostic stool samples. We identified 3 fecal biomarkers, alpha-2-macroglobulin (A2MG), matrix metalloproteinase-7 (MMP-7), and alpha-1-antitrypsin (A1AT), that were specific to CDI disease status. Additionally, these biomarkers correlated fairly with clinical severity. Importantly, select orthologs of this biomarker profile were also seen in a mouse model of CDI, implicating pathophysiologic congruency. The biomarkers presented here can enable the development of prognostic assays for CDI, performed concomitantly on diagnostic samples. More broadly, these markers provide unique insights into the immunopathogenesis of CDI complications and identify potentially novel therapeutic targets.

## Results

Two major goals of this research were to define (a) a core host defense profile that is specific for CDI and (b) host defense signatures that biologically segregate disease severity and correlate with clinical outcome. This study involved 2 key initial components: (a) developing a top-down proteomics approach to identify a range of human proteins/peptides from stool and (b) screening of stool samples and cohort building for the discovery of CDI fecal biomarkers using the developed method.

### Top-down proteomic analysis of human stool.

A top-down proteomics workflow was established for proteoform profiling of human stool. The term “proteoform” is synonymous to protein isoform and refers to any of the different protein products of a single gene, including changes due to genetic variations, alternative splicing, and posttranslational modifications (18). Protein extraction, sample preparation, LC-MS/MS, and data analysis methods were rigorously optimized as described in *Proteomics analysis* in Supplemental Methods; supplemental material available online with this article; https://doi.org/10.1172/jci.insight.142976DS1

### Sample characteristics and meta-analysis.

Stool samples were prospectively collected and tested for CDI in the diagnostic microbiology laboratory of the MUHC, using a commercial, FDA-cleared real-time PCR diagnostic platform targeting *C*. *difficile* toxin B (*tcdB*), with an established negative predictive value of at least 99%. Diarrheal samples included only those in which stool was unformed or liquid (Bristol Stool chart types 5 to 7; see *Clostridioides difficile infection diagnosis* in Supplemental Methods): CDI-negative (*N* = 49; 24 F; 25 M; 66 ± 4 years old) and CDI-positive (*N* = 54; 26 F; 28 M; 62 ± 5 years old) diarrheal samples were obtained. In addition, nondiarrheal stool samples from healthy controls and confirmed CDI-negative subjects were obtained (*N* = 8). No correlation was found between sex or age and stool protein concentration (BCA protein assay) or number of identified proteoforms (Supplemental Table 1). However, CDI-negative stool samples were more consistent (less fluid) and had more protein content (mg/mL) compared with CDI-positive samples; however, more proteoforms were identified in the latter relative to the negative controls. There was also moderate negative correlation (Pearson’s *r* = –0.51) between stool consistency and protein concentration; i.e., more consistent stool had a lower protein concentration on average. No significant correlation was found between stool consistency and number of proteoforms or number of proteoforms and protein concentration (Supplemental Figure 1). Likewise, no significant difference was found in stool consistency or number of proteoforms in the patients with gastrointestinal bleeding (*N* = 4). Bloody stool had a higher fecal protein concentration compared with normal stool (Supplemental Figure 2, A–C).

### Fecal biomarkers of CDI in humans.

**Figure 1:**
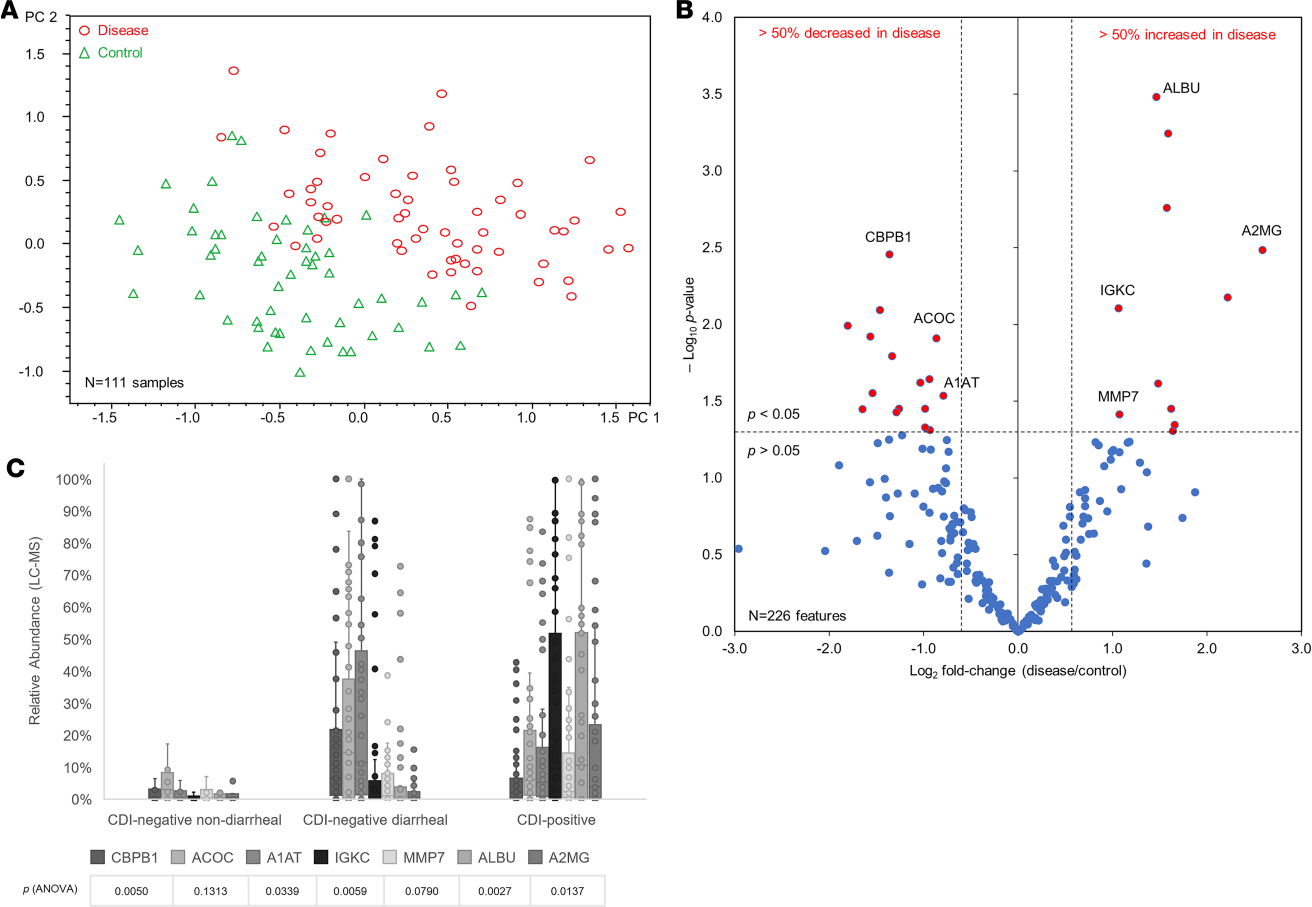
Fecal host biomarker discovery of CDI by top-down proteomics. (**A**) Partial least squares regression plot with 95% Pareto scaling of CDI-positive (disease; *N* = 54) and CDI-negative (control; *N* = 57) stool samples. (**B**) Volcano plot illustrating significantly (*P* < 0.05) differentially expressed (fold change ≥ ±1.5) proteoforms in disease samples. (**C**) Relative abundance (determined by LC-MS signal intensity) of identified candidates in nondiarrheal CDI-negative (*N* = 8) and diarrheal CDI-negative (*N* = 49) and CDI-positive (*N* = 54) samples. Error bars represent 95% confidence interval. Significance levels were assessed by 1-way ANOVA and the *F* test.

Partial least squares (PLS) analysis of samples (*N* = 111) demonstrated that they comprised statistically distinct constituents (i.e., proteoforms) (Figure 1A). A total of 226 proteoforms within 1.0–100.0 kDa were detected in the analyzed samples with a signal-to-noise ratio > 5 and quality factor > 0.65. Twenty-seven proteoforms had significantly altered levels in patients with CDI (11 overabundant and 16 underabundant in the patients), from which 7 proteins were identified by targeted LC-MS/MS (Figure 1B). The remaining proteoforms either were nonhuman or did not yield confident protein search results; i.e., their protein scores (Mascot) were less than 20. The identified proteins that were increased in patients with CDI were A2MG, serum albumin (ALBU), MMP-7 , and Ig kappa constant (IGKC). The ones that were decreased were carboxypeptidase B (CBPB1), cytoplasmic aconitate hydratase (ACOC), and A1AT. These potential biomarkers were statistically significant (*P* ≤ 0.03) and highly confident (protein score = 190 ± 100; mean ± SEM) proteins that could distinguish patients with CDI from negative controls with fair/good selectivity and specificity (receiver operating characteristic [ROC] AUC = 0.7–0.9) (Table 1 and Supplemental Figure 3). Two biomarkers (CBPB1 and A1AT) did not distinguish between nondiarrheal samples and CDI. However, they were significantly different (*P* ≤ 0.01 and *P* ≤ 0.05, respectively) between non-CDI diarrhea and CDI groups; hence, they were CDI specific only if patients had diarrhea. In contrast, IGKC, ALBU, A2MG, and MMP-7 not only had increased levels in diarrheal samples but also were specific biomarkers of CDI. A2MG and CPBP1 had the highest fold changes in patients with CDI (6.00 and –2.57, respectively) (Table 1 and Figure 1C). Pearson’s correlation analysis revealed that ALBU levels had a strong positive relationship with IGKC (*r* = 0.91). ALBU also moderately positively correlated with MMP-7 (*r* = 0.61), while CBPB1 had a fair positive relationship with ACOC (*r* = 0.46). No other relationship was found between biomarker levels and patient characteristics or clinical parameters (Supplemental Table 2).

**Figure 2:**
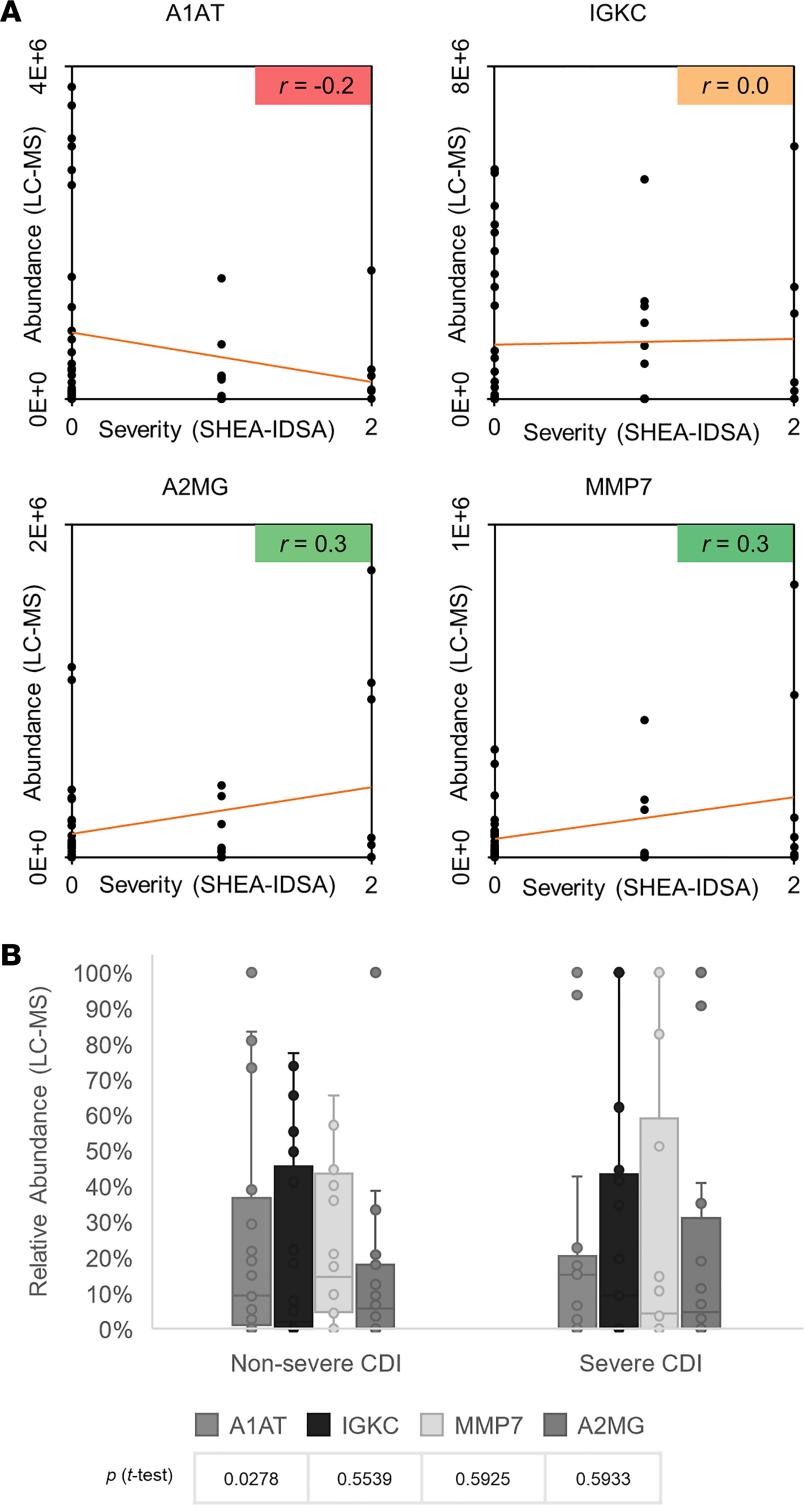
Correlation between CDI host biomarkers and disease severity. (**A**) Correlation plots for selected biomarkers in all CDI-positive samples for which a clinical score could be calculated (*N* = 50, outliers removed at α = 0.05). SHEA/IDSA scores 0, 1, and 2 were assigned to nonsevere, severe, and fulminant cases, respectively. Pearson’s correlation coefficient (*r*) between biomarker abundance (determined by LC-MS/MS signal intensity) and CDI severity (SHEA/IDSA) was assessed. (**B**) Biomarker abundances in patients with nonsevere and severe CDI. A1AT decreased with CDI severity while MMP-7 and A2MG increased with CDI severity. IGKC did not correlate with CDI severity. Error bars represent 95% confidence interval. Significance levels were assessed by Student’s *t* test.

CDI severity, based on SHEA/IDSA criteria (3), could be assessed for 50 of the 54 CDI-positive patients. Correlation analysis showed that 3 biomarker candidates correlated with CDI severity. A2MG and MMP-7 had positive (*r* = 0.3), A1AT had negative (*r* = –0.2), and IGKC had no correlation (*r* = 0.0) with disease severity (Figure 2). Moreover, while IGKC and ALBU were more abundant (*P* > 0.05) in patients with gastrointestinal bleeding, IGKC and A1AT increased (*P* > 0.05) with CDI progression (Supplemental Figure 2, D–E).

### Fecal biomarkers of CDI in mice.

**Figure 3:**
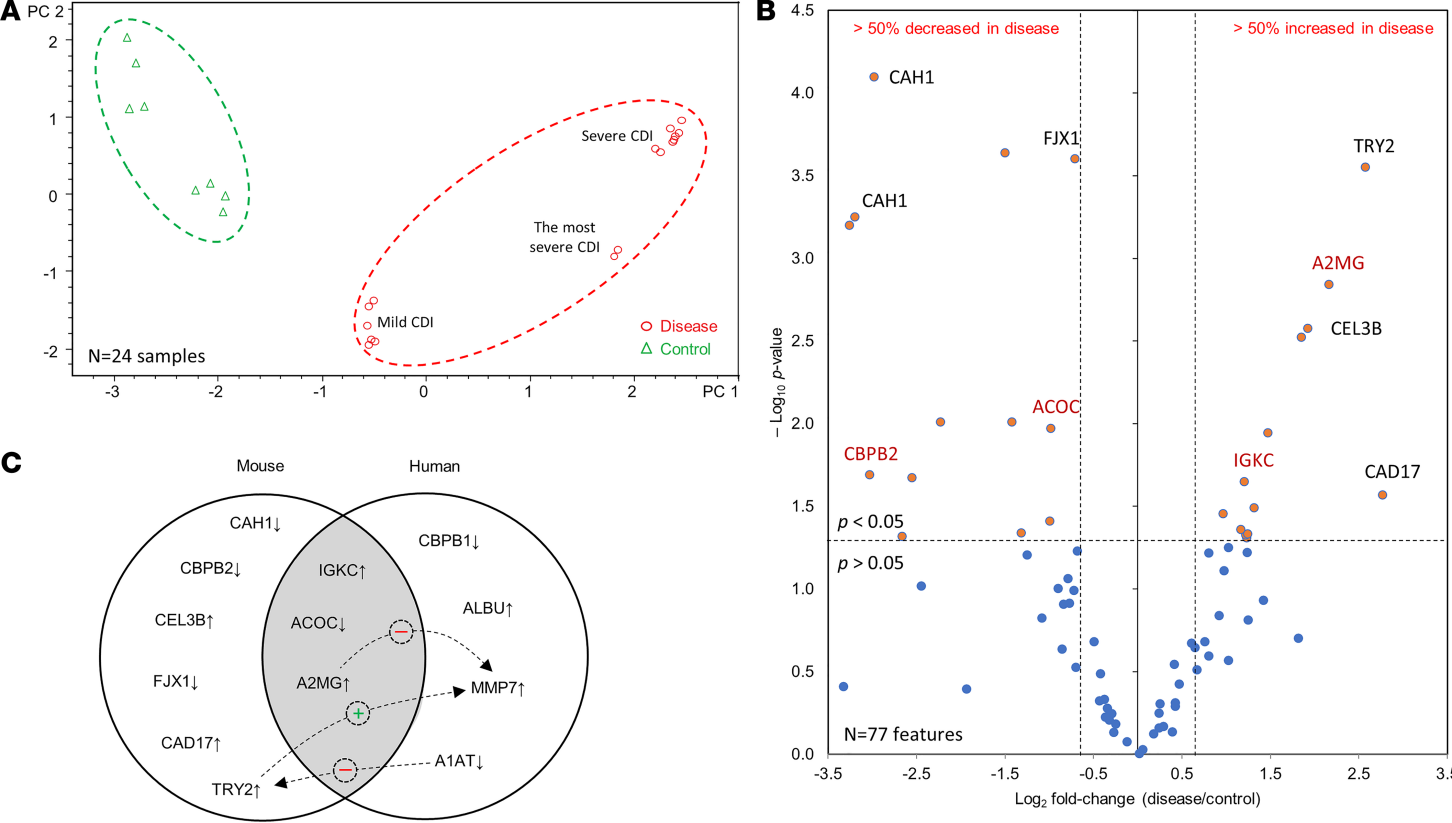
CDI biomarker confirmation in mice. (**A**) Principal component analysis plot with 95% Pareto scaling of CDI-positive (disease; *N* = 8) and CDI-negative (control; *N* = 4) mice. The analysis was performed in duplicate. (**B**) Volcano plot illustrating significantly (*P* < 0.05) differentially expressed (fold change ≥ ±1.5) proteoforms in disease samples. Those also identified in humans are marked in red. (**C**) Venn diagram demonstrating potential links between CDI biomarkers identified in mice and humans.

We used an established mouse model of CDI (19, 20) to determine the pathophysiologic relevance of the identified human biomarkers: mice were challenged with no, low-dose, or high-dose *C*. *difficile* to mimic different severity of CDI (see *Mouse experiment* in Supplemental Methods for details). The murine experiment revealed that LC-MS/MS data acquired from *C*. *difficile*–infected and uninfected mice formed separate groups, similar to human data. Principal component analysis (PCA) separated the mice (*N* = 24) with mild and severe CDI, i.e., infected with low- or high-dose *C*. *difficile*, respectively (Figure 3A). From the 77 proteoforms detected within 1.0–100.0 kDa, 26 had significantly different levels in the feces of infected mice (13 over- and 13 underabundant in CDI), out of which 9 host proteins were identified. Four murine proteins were identified, corresponding to the potential CDI human biomarkers: A2MG, IGKC, ACOC, and CPBP2 (an ortholog of human CPBP1 with 41% homology; UniProt). Similar to humans, CPBP2 and ACOC were decreased, whereas IGKC and A2MG were increased in the feces of CDI mice (Figure 3B). Other proteins that were decreased in CDI mice were carbonic anhydrase 1 (CAH1) and four-jointed box protein 1 (FJX1). Chymotrypsin-like elastase 3B (CEL3B), anionic trypsin-2 (TRY2), and cadherin-17 (CAD17) were increased. CAD17 and CPBP2 had the most drastic changes in CDI mice with 6.80- and –8.18-fold changes, respectively (Table 2). Physiologic relatedness between CDI biomarkers identified in mice and humans is depicted in Figure 3C.

Pathway overrepresentation analysis (ORA) (21) based on the identified biomarkers in humans revealed that 10 pathways were significantly (*P* < 0.05) altered in patients with CDI (Reactome). The 3 most affected pathways (highest number of altered genes) were (a) high-density lipoprotein-mediated lipid transport, (b) lipoprotein metabolism, and (c) lipid digestion, mobilization, and transport (Supplemental Table 3). The same analysis in mice led to 3 overrepresented pathways, including extracellular matrix (ECM) degradation, ECM organization, and innate immune system, 2 of which (ECM degradation and organization) were also perturbed pathways in humans (Supplemental Table 4). An interaction analysis uncovered 361 protein-protein, 18 protein-DNA, and 1 DNA-DNA interactions among human biomarkers. Most of the identified interactions involved physical association (*N* = 248) or physical interaction (*N* = 103). ALBU, A2MG, and A1AT accounted for 93% of the identified interactions (Supplemental Figure 4 and Supplemental Table 5).

### Confirmation of identified biomarkers using antibody-based detection.

**Figure 4:**
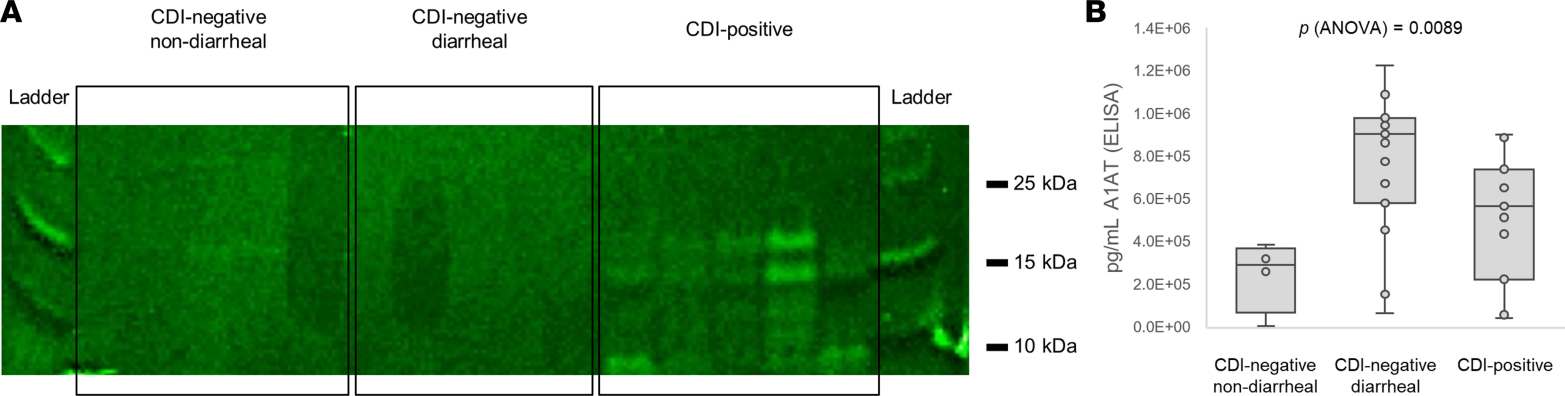
Orthogonal confirmation of selected biomarkers of CDI by antibody-based methods. (**A**) Immunoblot of IGKC (5 nondiarrheal CDI-negative; 4 diarrheal CDI-negative; 5 CDI-positive) and (**B**) ELISA of A1AT (5 nondiarrheal CDI-negative; 15 diarrheal CDI; 11 CDI-positive). The box plots depict the minimum and maximum values (whiskers), the upper and lower quartiles, and the median. The length of the box represents the interquartile range. Error bars represent 95% confidence interval. Significance levels were assessed by 1-way ANOVA and *F* test.

Biomarker candidates that were biologically relevant to host defenses were selected for orthogonal confirmation by antibody-based methods. Immunoblot analysis of randomly selected negative and positive samples (*N* = 10) confirmed that IGKC was overabundant in the stool of patients with CDI (Figure 4A). Likewise, A1AT measurement by enzyme-linked immunosorbent assay (ELISA) in 30 human samples yielded results similar to those obtained by LC-MS/MS; i.e., A1AT had higher levels in diarrheal samples compared with stool from healthy controls; however, among diarrheal samples, A1AT was significantly diminished in those from patients with CDI (Figure 4B and Figure 1D). Other biomarker candidates could not be detected by Western blot.

## Discussion

To distinguish a unique host-based protein signature for CDI, we established the necessary infrastructure for a top-down proteomics method to identify a range of human proteins extracted from stool. In brief, we developed a robust and reproducible method for sample processing, i.e., protein extraction from stool, and proteoform profiling of fecal extracts, including the detection, statistical assessment, and orthogonal confirmation of potential host-derived biomarkers of CDI. To ensure that we could detect the broadest range of human proteins needed for this study, we optimized yield of proteoform detection by reiterative assessment of various analytical approaches (*Proteomics analysis* in Supplemental Methods). In parallel, we established 4 cohorts of adult subjects, defined as follows: (a) nondiarrheal non-CDI controls; (b) diarrheal non-CDI controls; (c) CDI nonsevere; and (d) CDI severe, with severity based on an established SHEA/IDSA scoring system. With this approach, we were able to demonstrate that CDI samples statistically clustered distinctly from non-CDI controls. Statistical analysis identified 27 biomarker candidates that were significantly differentially (up-/down-) recovered from samples; 7 were human proteins. A2MG and MMP-7 fairly positively correlated with clinical severity, while A1AT negatively did so, suggesting that these biomarkers are potentially indicative of CDI severity. The latter biomarker was confirmed in selected stool samples by ELISA-based immunodetection. Four candidates were also found in the CDI mouse model. The murine orthologs of A2MG and IGKC were identified. While the orthologs of MMP-7 and A1AT were not identified with statistically significant differential expression, TRY2 was found to be significantly elevated in CDI mice (Table 2). TRY2 is known to form a complex with its inhibitor, A1AT (22), while TRY2 is a potent activator of MMPs, including MMP-7 (23). Thus, despite differences in protein detection that may reflect species-specific differences (24), the identified molecules in response to CDI are physiologically related, suggesting a common pathway of immunopathogenesis in CDI.

The biomarkers confirmed in this study have biological functions that could be associated with CDI etiology. A2MG and A1AT are serum protease inhibitors primarily synthesized in the liver involved in numerous biological processes. A1AT is a serine protease inhibitor that protects tissues from enzymes of inflammatory cells, particularly neutrophil elastase, and therefore its concentration rises dramatically in response to acute inflammation (25). Persistent elevation of fecal A1AT has been linked to acute and chronic diarrhea in infants (26, 27). Our study confirmed that, in the presence of diarrhea, A1AT could separate CDI from non-CDI patients.

A2MG binds proinflammatory cytokines, such as tumor necrosis factor-α and interleukin-1β, and may be important in regulating chronic inflammation (28). Another important function of A2MG is regulating ECM homeostasis through inhibition of MMPs (29). CDI affects ECM production and stability in the intestine both directly and indirectly. *C*. *difficile* utilizes a highly immunogenic surface-associated cysteine protease to degrade ECM during the colonization process (30). Meanwhile, *C*. *difficile* exotoxins hamper epithelial cell–ECM interactions to increase the adherence of clostridia to target cells (31). MMPs are the main group of enzymes responsible for ECM degradation. Abnormal ECM expression and ECM fragmentation induced by tissue-remodeling processes can influence immune cell activation and survival in chronically inflamed tissues (32). Pathway ORA showed that ECM degradation and ECM organization were altered pathways in CDI in both humans and mice. The increase in fecal A2MG could be a response to excessive degradation of intestinal ECM in CDI. Our data suggest that A2MG is a CDI biomarker that segregates CDI patients from nondiarrheal and diarrheal non-CDI controls and that fairly correlates with disease severity in both humans and mice.

MMP-7 is produced by mucosal epithelium (33). Among its myriad of functions, MMP-7 can activate α-defensins, regulate intestinal permeability (34), promote re-epithelialization, and control migration of neutrophils into intestinal mucosa (35). MMP-7 also degrades collagen types III, IV, V, IX, X, and XI (36), which is pertinent because the intestinal ECM is primarily composed of collagen types I, III, and V (37). We detected an increased level of fecal MMP-7 in patients with CDI, which may contribute to disease severity via destruction of the intestinal ECM. MMP-7 was not found in CDI mice. However, TRY2 was: TRY2 has been shown to activate MMPs, notably MMP-7 (38, 39), and thus may reflect the murine physiologic ortholog of the identified human MMP-7 marker. TRY2 also forms a complex with A1AT (40), another CDI biomarker that was only detected in humans. Our findings suggest that MMP-7 is a host biomarker for CDI and that it can distinguish among non-CDI, nonsevere CDI, and severe CDI patients. Interestingly, doxycycline has long been recognized as one of the antibiotics with the least tendency to predispose to CDI (41). Although the mechanism for this protective effect has remained elusive, and may be partly related to direct effects (antimicrobial activity on *C*. *difficile*) or indirect effects (less disruption on gut microbiota than other antibiotics) (41), it is noteworthy that doxycycline is a known inhibitor of MMP-7 (42, 43). Our findings may provide insight into this heretofore-unexplained effect of doxycycline in CDI.

IGKC is the constant region of Ig light chains. It has been shown that the light chains of IgG, IgM, and IgA bind nonspecifically to *C*. *difficile* toxin A in mice, although the consequences of this binding were not determined (44). Based on our LC-MS/MS data, fecal IGKC is doubled in response to CDI in both humans and mice. In humans, it was able to segregate nondiarrheal, non-CDI diarrheal, and CDI samples, suggesting that it is specific to CDI. However, it did not correlate with CDI severity.

Other potential biomarkers of CDI in humans were ALBU, ACOC, and CBPB1. ALBU is a predictor of CDI recurrence, complications, and mortality (45). Hypoalbuminemia is usually associated with severe CDI (46). We did not find any correlation between fecal ALBU and CDI severity or between the fecal and serum levels of ALBU. However, it was more abundant in the feces of the patients with gastrointestinal bleeding potentially because of the high abundance of ALBU in human blood. ACOC regulates uptake, sequestration, and utilization of iron when cellular iron levels are low and was the only intracellular CDI biomarker candidate found in both humans and mice. Increased colonic iron favors the abundance of enteropathogenic bacteria in preference to beneficial barrier commensal gut bacteria (47). Iron is essential to the growth and pathogenicity of *C*. *difficile* (48). Decreased ACOC concentration in CDI stool could be part of the host response to the infection. CBPB1 is a metallocarboxypeptidase and a key regulator of fibrinolysis and tissue inflammation (49). Increased pancreatic secretion of CBPB1 has been associated with diarrhea in piglets (50). We detected higher CBPB1 levels in non-CDI diarrhea compared with both CDI and healthy (nondiarrheal) controls. Indeed, CBPB1 and its mouse ortholog (CBPB2) were the most strongly decreased biomarkers in CDI stool relative to non-CDI controls, suggesting they could efficiently separate CDI from other diarrheal etiologies.

A major limitation of the present approach is its limited detection capacity for small-size biomarkers (molecular weight [MW] < 5 kDa). Top-down proteomics generally improves protein detection efficiency by eliminating the protein digestion step that drastically increases sample complexity. However, small proteins and oligopeptides either do not retain efficiently on the chromatography columns commonly used in proteomics experiments or poorly ionize during the MS analysis and, hence, remain undetected or are masked under more abundant background contaminants. Moreover, although earlier studies show that fecal cytokines correlate with disease severity (51, 52), we did not identify any cytokine biomarker in CDI stool. Generally, the application of MS to cytokine analysis is challenging. Cytokines are low-abundance proteins with endogenous concentrations in the fg/mL to pg/mL range, which is below the detection limit of most MS instruments (53). While it is theoretically possible to detect cytokines in small sample quantities using MS, their detection suffers greatly from matrix interferences and/or suppression (54). Similarly, the analytical sensitivity of immunoblot using commercially available antibodies is generally weaker than that of MS-based proteomics, likely accounting for the difficulty in orthogonal validation of the biomarkers.

In conclusion, 3 host biomarkers that correlated with disease status and potentially with clinical outcome were identified for CDI in human stool using a novel, robust, and reproducible approach. The knowledge gained from this research could provide significant insights into host mechanisms of protective and pathologic responses to CDI. These data, derived from stool and reflecting host intestinal responses, are highly relevant and novel; the congruency in the composition and the magnitude of responses observed between humans and mice reflect a unique, *C*. *difficile*–specific host defense program. Further studies are needed to confirm the pathophysiologic role of these biomarkers and to map the host responses that fundamentally regulate CDI and its progression. The fecal biomarker profile that we have shown to segregate with CDI and possibly with disease severity will require validation in other cohorts but may provide novel targets for prognostic assays and for therapy of CDI.

## Methods

Experimental details can be found in Supplemental Methods.

### Sample population and cohorts.

Stool samples were collected from 111 adult individuals (age ≥ 18 years) in 3 cohorts as follows: 54 patients with CDI, evaluated clinically for the presence of diarrheal syndrome and with positive diagnostic testing for *C*. *difficile*; 49 non-CDI diarrheal patients, screened clinically for the presence of diarrheal syndromes but with negative diagnostic testing for *C*. *difficile*; and 8 healthy controls (nondiarrheal non-CDI), screened clinically for absence of diarrheal syndrome and diagnostically with negative test for *C*. *difficile*. Severity was graded based on SHEA/IDSA criteria (3).

### Top-down proteomics analysis.

Fecal matter was homogenized in a chaotropic buffer system in the presence of protease inhibitors to prevent proteolytic degradation, filtered to remove submicron particles, and cleaned using reverse-phase (C_4_) solid-phase extraction prior to LC-MS/MS analysis. Proteoform profiling was performed by a Bruker Maxis II quadrupole time-of-flight tandem mass spectrometer in positive electrospray ionization mode using the Sophisticated Numerical Annotation Procedure algorithm (55) for molecular species with a MW of 1.0–100.0 kDa and a maximum charge state of 40+. Proteoforms present in at least 60% of the samples within each cohort elucidating at least ±50% change (*P* < 0.05, Student’s *t* test) in signal intensity between the cohorts with an ROC AUC ≥ 0.6 were selected for targeted MS/MS analysis using collision-induced dissociation (99.5% N_2_). Protein identification was conducted by top-down protein sequencing against the human subset of UniProt/Swiss-Prot protein database. Identified proteins were confirmed in randomly selected patients (*N* = 10–30) by antibody-based detection. LC-MS/MS raw data from this analysis have been deposited to the ProteomeXchange Consortium via the PRoteomics IDEntifications partner repository (56) with the data set identifier PXD020117.

### Murine infection.

The CDI mouse model was established as previously described (19, 20). Details are provided in *Mouse experiment* in Supplemental Methods. Briefly, 8- to 11-week-old, antibiotic-treated, male C57BL/6J mice were given 0 (control), 2.32 × 10^5^ (low dose), or 1.62 × 10^6^ (high dose) CFU/mouse of freshly cultured *C*. *difficile* strain VIP10463 (ATCC 43255) by gavage. In previous studies, the high dose generated a lethal infection, and the low dose generated a sublethal infection (20). Mice were then monitored and scored for weight loss, activity, posture, coat quality, diarrhea, and eye and nose symptoms (57). Twenty-four hours after infection mice were euthanized by isoflurane-CO_2_; fecal matter was collected from the cecum and colon and stored in the protein solubilization cocktail described earlier for proteomics analysis. The mouse experiment was approved by the Animal Care Committee of McGill University and performed in accordance with the guidelines of the Canadian Council on Animal Care.

### Orthogonal confirmation.

Fecal protein was denatured and separated by SDS-PAGE. Protein bands were transferred to a nitrocellulose membrane for immunoblot detection. Membranes were blocked with bovine serum albumin and incubated with antibodies against IGKC (clone HP6053-L1C1), human serum albumin (clone 188835), A2MG (clone 257316), ACOC/ACO1 (clone EPR7225), A1AT/SerpinA1 (clone 202808), CBPB1 (clone 438806), or MMP-7 (clone 111433); all antibodies were purchased from R&D Systems, Bio-Techne. Rinsed membranes were then incubated with horseradish peroxidase–conjugated secondary IgG (GE Healthcare) and protein detected by chemiluminescence. A1AT was also detected by sandwich ELISA to human SerpinA1 (R&D Systems, Bio-Techne; catalog DY1268) following the manufacturer’s instructions.

### Statistics.

Statistical analysis was performed in Compass ProfileAnalysis software (Bruker). Proteoforms with *m/z* 1500–30,000 were subjected to Student’s 2-tailed heteroscedastic *t* test. Candidates elucidating at least ±50% change (*P* < 0.05) in LC-MS/MS signal intensity between the cohorts were selected for protein identification. PCA and PLS regressions (Pareto scaling, 95%) were performed to evaluate the discriminatory power of the proteomic model, whereas ROC analysis was employed to assess the sensitivity and specificity of each biomarker candidate. Statistical significance was evaluated between the cohorts using 1-way ANOVA and *F* test. Pathway ORA (hypergeometric algorithm and Benjamini-Hochberg correction) and protein-protein interaction analysis were performed by InnateDB integrated analysis platform (21) with UniProt identifiers as the cross-reference database. *P* < 0.05 was considered significant.

### Study approval.

The human study was reviewed and approved by the MUHC Research Ethics Board (REB), Montréal, Québec, Canada (protocol 2017-1785), and US Army Medical Research and Materiel Command’s Office for Human Research Protections (Human Subjects Research Review Board Log Number A-19360). Patients were recruited between 2016 and 2017 at the MUHC, Montréal, Québec, Canada. All patients provided informed consent prior to their participation in the study. The mouse study was conducted under the RI-MUHC–approved animal use protocol 7797 (Animal Care Committee of McGill University).

## Author contributions

DCV conceived the study. DCV and MN were in charge of overall direction and planning. MG conducted the proteomics analysis, analyzed the data, and composed the manuscript. KW performed the murine experiment. DCV, MeL, and VGL conducted clinical and microbiologic analyses. MeL collected the clinical data and stool samples and obtained informed consent from the patients. MaL and LR conducted the orthogonal confirmation experiments. DCV and MG wrote and revised the manuscript.

## Supplementary Material

Supplemental data

Supplemental Table 5

## Figures and Tables

**Table 1:**
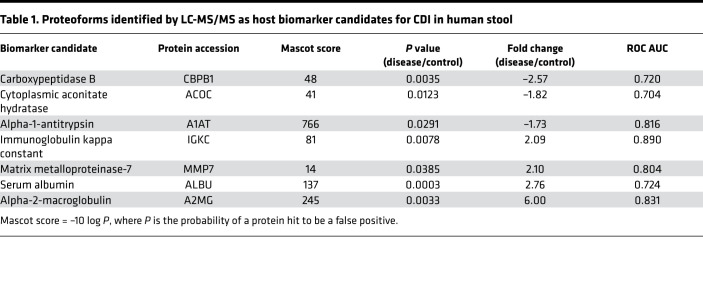
Proteoforms identified by LC-MS/MS as host biomarker candidates for CDI in human stool

**Table 2:**
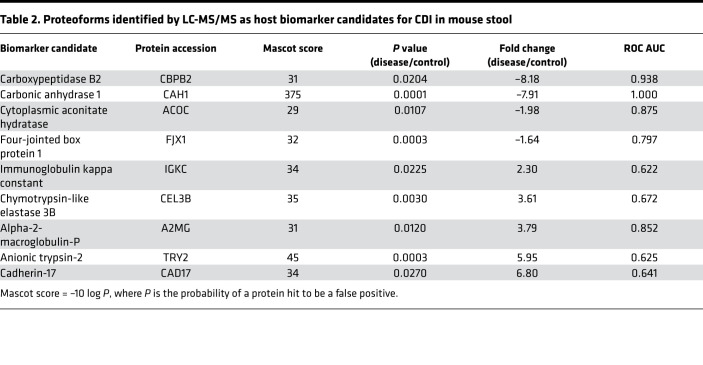
Proteoforms identified by LC-MS/MS as host biomarker candidates for CDI in mouse stool

## Version 1 11/24/2020

In-Press Preview

## Version 2 01/11/2021

Electronic publication
