# Supplementary material for: Fecal host biomarkers predicting severity of Clostridioides difficile infection
Source: JCI Insight. 2021 Jan 11;6(1):e142976. doi: 10.1172/jci.insight.142976 (PMC7821589; doi:10.1172/jci.insight.142976)
Supplement: Supplemental data [file jciinsight-6-142976-s007.pdf]

Fecal host biomarkers predicting severity of *Clostridioides difficile* infection

Makan Golizeh[1], Kaitlin Winter[1,2], Marija Landekic[1,2], Lucie Roussel[1], Mélanie Langelier[1], Vivian G. Loo[1,2,3], Momar Ndao[1,2,3,] and Donald C. Vinh[1,2,3,]

[1] *Infectious Diseases and Immunity in Global Health (IDIGH) Program, Research Institute of the McGill University Health Centre (RI-MUHC), Montreal, Quebec, Canada*

[2] *Department of Microbiology and Immunology, McGill University, Montreal, Quebec, Canada*

[3] *Department of Medicine, McGill University, Montréal, Québec, Canada*

Corresponding authors: Momar Ndao, momar.ndao@mcgill.ca; Donald C. Vinh, donald.vinh@mcgill.ca.

Supplemental Information

Index

Section	Page
**S1** Proteomics analysis …………………………………………………………………	2
**S2** *Clostridioides difficile* infection diagnosis ………………………………………………	4
**S3** Mouse experiment ………………………………………………………………………	4
**S4** Immunoblotting procedure …………………………………………………………	5
References for Supplemental information…………………………………………………..	6
**S5** Supplemental Figures ………………………………………………………………...	7
**S5** Supplemental Tables ………………………………………………………………………..	11

S1. Proteomics analysis

Fecal material (~ 1.0 g) was transferred into a sterile low protein binding 1.5-mL plastic tube and suspended in 0.5 mL 8 M urea, 0.1 M NaCl, 0.025 M Tris pH 8.2 solution containing Pierce protease inhibitor (Thermo Fisher Scientific, Waltham, MA). Samples were mixed vigorously (4 x 30 s) and centrifuged (2500 g, 5 min). The supernatant was pipetted into a clean tube and re-centrifuged (20,000 g, 30 min). The supernatant was then filtered on a Pierce ~30 μm polyethylene centrifugal filter (8000 g, 10 min) and re-filtered using a Corning Spin-X 0.45 μm cellulose acetate centrifugal filter (Thermo Fisher Scientific, 12,000 g, 15 min). The filtrate (0.15 mL, 0.4 mg total protein, quantified by Pierce BCA protein assay kit) was loaded on to a re-hydrated reverse-phase C_4 solid-phase extraction (SPE) spin column (Harvard Apparatus, Holliston, MA). The unbound fraction was collected (2000 g, 4 min) and discarded. C_4 material-bound species were eluted from the column (2000 g, 4 min) with acetonitrile (ACN, 1% formic acid) and evaporated to dryness under vacuum (2 h, 37°C). Samples were kept at ≤ 4°C during processing.

Liquid chromatography mass spectrometry (LC-MS) analysis was performed on a Dionex Ultimate 3000 UHPLC system (Thermo Fisher Scientific) coupled to a Maxis II high-resolution quadrupole-time of flight (Q-TOF) tandem mass spectrometer (Bruker, Billerica, MA) equipped with an Apollo electrospray ionization (ESI) source (Bruker). Briefly, the cleaned-up fecal protein extract (**Section S1.2**) was reconstituted in 1% formic acid (60 μL) and injected (20 μL) on to a Jupiter C_4 1 x 150 mm 5 μm (300 Å) HPLC column (Phenomenex, Torrance, CA) and separated using a gradient elution with ACN (0.8% formic acid) in ultra-pure water (1% formic acid) at 125 μL/min (50°C). % ACN was at 5% within the first 4 min, during which sample was being loaded on to the column, then increased to 20% at 10 min, to 50% at 30 min, to 70% at 40 min, to 90% at 45 min, held for 5 min, and then changed back to 5% to re-equilibrate the column for an additional 10 min. MS analysis was conducted in positive ionization mode with a source voltage of 4.5 kV for the mass-to-charge ratio (*m/z*) of 400–3000 with an acquisition rate of 1.00 Hz. A continuous flow of 99.5% pure N_2 was provided (Parker, Mayfield Heights, OH, USA) as dry gas (200°C) at a flow rate of 6 L/min. A low-concentration ESI tuning mix (Agilent Technologies, Santa Clara, CA) was used for post-column infusion external calibration within the first 3 min of every LC-MS analysis. Samples were analyzed in random order in the sequence to avoid biases due to the injection order and one blank (5 μL 1% formic acid in water) was inserted between each two analyses to minimize carryover.

Proteoform detection was conducted by Compass DataAnalysis software (Bruker) using Sophisticated Numerical Annotation Procedure (SNAP) algorithm [1] for LC-MS features within a MW range of 1.0–100.0 kDa and a maximum charge state of 40+. The signal-to-noise (S/N) and quality factor thresholds were set to 5.0 and 0.65, respectively. Data processing was performed on a Hewlett-Packard PC

workstation (Palo Alto, CA) equipped with an Intel (Santa Clara, CA) Xenon 8-core 3.70 GHz CPU and 16 GB RAM. The list of proteoforms was imported into the Compass ProfileAnalysis software (Bruker) for statistical analysis. The software generated a list of biomarker candidates with *m/z* 1500–30,000 and retention time 10–55 min present in at least 60% of the samples within each cohort using the time alignment option to account for LC retention shifts. Candidates elucidating ≥ ±50% change ($p < 0.05$, Student's *t*-test) in signal intensity between the cohorts were selected for protein identification by liquid chromatography tandem mass spectrometry (LC-MS/MS). To this end, samples with relatively high abundance of each candidate were re-analyzed (20 µL) using the LC parameters employed in the discovery phase coupled with a targeted MS/MS method to selectively fragment the biomarker candidate at the given *m/z* value and retention time. The MS/MS analysis was performed at an acquisition rate of 1.08 Hz using collision-induced dissociation. LC-MS/MS data were processed in DataAnalysis software and the MS/MS spectra associated with selected biomarker candidates were sent to BioTools software (Bruker) for protein identification using Mascot search engine (Matrix Science, London, UK) against the UniProt/Swiss-Prot protein database (downloaded on 28 July 2017; 555,100 sequences). Top-down sequencing was conducted by adding methionine oxidation and asparagine/glutamine deamidation as variable modifications with a mass tolerance of 50 ppm and 50 mDa at the MS and MS/MS levels, respectively. Other statistical testing including principal component analysis (PCA), partial least squares (PLS) and receiver operating characteristic (ROC) analysis were performed by the ProfileAnalysis software (Bruker).

Gene ontology information was obtained from the UniProt knowledgebase. Pathway over-representation analysis (hypergeometric algorithm & Benjamini-Hochberg correction) and protein-protein interaction analysis were performed by InnateDB integrated analysis platform [2] with UniProt identifiers as the cross-reference database. The results were subsequently visualized with the Cerebral plugin [3] of Cytoscape software to organize the interaction network in the context of subcellular localization for all of the proteins displayed and visualized by Cerebral.

The LC-MS method was optimized for proteins within 1.5–80.0 kDa using an intact protein mix standard solution (Sigma-Aldrich). The limit of detection (LOD) was determined using 0.1–1.5 mg/mL solutions from the intact protein mix. The LOD for proteins within the range of 5.0–30.0 kDa was found to be approximately 0.2–0.5 µg for the LC column used in this study. This LOD may be insufficient for the detection of some immunologically important but usually low-abundance fecal proteins such as calprotectin (50 µg/g feces) [4]. Low-end micro-flow and nano-flow LC-MS is known to ameliorate signal-to-noise ratio and thus method sensitivity via decreased analyte diffusion due to smaller column diameter and lower flow rate [5]. Initial tests on 0.3 mm and 0.075 mm i.d. HPLC columns at reduced

flow rates (6.0 and 0.3 µL/min, respectively) yielded promising results however the method failed to deliver reproducible results.

Initial tests also demonstrated that the peak finding script that employed SNAP algorithm occasionally failed to detect complex MS spectra associated with excessively charged high-MW proteins such as serum albumin (69.3 kDa) and transferrin (77.1 kDa). An alternate technique, maximum entropy (MaxEnt), has been successfully applied to deconvolute electrospray mass spectra of multiply charged proteins [6]. In contract to SNAP, MaxEnt is known for its effectiveness in the detection of super-complex MS spectra of larger proteins albeit at the expense of longer data processing time [6]. While a regular peak detection process using SNAP lasted 30–45 min, MaxEnt processing took 6–8 h to complete on the same computer. A hybrid SNAP/MaxEnt method did not considerably improve peak detection efficiency. However, excluding known primary contaminants, such as common detergents and polymeric species, from the MS acquisition method led to faster generation of a cleaner LC-MS peak list.

S2. *Clostridioides difficile* infection diagnosis

Stool samples were submitted by the treating physician on their real-world, clinical suspicion for CDI. Blood-based laboratory parameters were measured by the treating physicians as deemed necessary by them and retrospectively analyzed to grade severity as per SHEA/IDSA criteria. Diagnostic testing for *C. difficile* was performed on stool using the standard operating procedure of the clinical diagnostic microbiology laboratory of the McGill University Health Centre. In particular, the acceptation and rejection criteria for stool form is based on the Bristol stool chart [7], such that stool samples acceptable for testing are unformed or liquid (i.e. Bristol types 5 to 7, inclusive), while other stool forms are not tested. Testing is performed on a commercial, FDA-cleared, real-time PCR diagnostic platform targeting the *C. difficile* tcdB gene, with an established negative predictive value of ≥ 99%. The medical charts of all subjects were reviewed independently by clinical health care workers to ensure that the criteria of CDI were met (diarrhea and either a stool test positive for *C. difficile* toxins, or colonoscopic or histopathologic findings revealing pseudomembranous colitis [8]), in the absence of any other cause for diarrhea (e.g. iatrogenic, including laxative or feeding solutions), and that positive *C. difficile* results were treated. Of 54 patients with CDI, 50 had blood-based parameters measured within 24 hours of their diagnostic stool sample, allowing for assessment of their CDI severity.

S3. Mouse experiment

C. difficile Strain VPI10463 (ATCC 43255) was obtained from Cedarlane Labs (Burlington, ON). Bacteria were maintained in meat broth (Sigma-Aldrich, Saint Louis, MO) containing 0.1% (w/v) L-cysteine in an anaerobic jar. For colony counts, *C. difficile*-containing media was serially diluted and streaked onto pre-reduced brain heart infusion plates (BD Biosciences, Mississauga, ON) containing 0.1%

L-cysteine. Plates were incubated at 37°C in an anaerobic jar overnight. Eight to eleven-week-old male C57BL/6J mice were given 2.15 μL/mL acetic acid in drinking water one week prior to antibiotic treatment. Six days prior to infection, mice were given an antibiotic cocktail in drinking water for 3 days. The cocktail included metronidazole (0.215 mg/mL), gentamicin (0.035 mg/mL), vancomycin (0.045 mg/mL), kanamycin (0.400 mg/mL) and colistin (0.042 mg/mL). Regular water was returned and, 24 hours prior to infection, mice were given clindamycin at 32 mg/kg based on the average mouse weight intraperitoneally, in 0.2 mL phosphate buffer saline. A 0.5-cc syringe with 28 G ½ needle was used for injection. Mice were given 0 (control; N=4), 2.32 x 10^5 (low dose; N=3) or 1.62 x 10^6 (high dose; N=5) cfu/mouse of *C. difficile* from fresh culture by gavage. Doses were estimated the day of infection using OD600 values and confirmed by colony counts. In previous studies, the high dose generates a lethal infection in mice and the low dose generates a sublethal infection [9]. During pre-treatment and infection, the groups were housed separately to avoid contamination between groups due to coprophagy. Twenty-four hours after infection, mice were euthanized with isoflurane anesthesia followed by CO_2 and subsequent cardiac puncture. The intestine was cut just above the cecum and at the anus. It was placed in clean Petri dish and the fecal matter was pushed out of the cecum and colon and stored in the protein solubilization cocktail described in **Section S1**. Samples were briefly spun (4000 g) and stored at –80°C until ready for use. LC-MS analyses were performed on each mouse in duplicate.

S4. Immunoblotting procedure

Fecal protein (25 μg) was denatured using NuPAGE reducing agent (Thermo Fisher Scientific) and separated on a NuPAGE 4–12% polyacrylamide Bis-Tris gel (200 V, 35 min, MES buffer). Protein bands were transferred to a nitrocellulose membrane using an iBLOT dry transfer system (Thermo Fisher Scientific) for immunoblot detection. Successful transfer was confirmed by Ponceau Red staining. Membranes were blocked (1 h) with 5% bovine serum albumin (BSA) in Tris-buffered saline, 0.05% Tween-20 (TBST), and incubated (4°C, overnight) with primary antibody (in 1% BSA/TBST v/v) followed by a second incubation (1 h) with horseradish peroxidase-conjugated anti-mouse or anti-rabbit IgG (1:15,000 v/v; GE Healthcare, Mississauga, ON). Membranes were developed by Pierce Super Signal West Pico chemiluminescence detection solution (Thermo Fisher Scientific) and exposed to radiography films for visualization. Primary antibody working solutions were prepared based on manufacturer's recommendations.

References

1. Rockwood AL, Orden SL Van. Ultrahigh-Speed Calculation of Isotope Distributions. Anal Chem. 1996; 68(13):2027–2030.

2. Breuer K, Foroushani AK, Laird MR, et al. InnateDB: systems biology of innate immunity and beyond—recent updates and continuing curation. Nucleic Acids Res. 2013; 41(D1):D1228–D1233.

3. Barsky A, Gardy JL, Hancock REW, Munzner T. Cerebral: a Cytoscape plugin for layout of and interaction with biological networks using subcellular localization annotation. Bioinformatics. 2007; 23(8):1040–1042.

4. Joshi S, Lewis SJ, Creanor S, Ayling RM. Age-related faecal calprotectin, lactoferrin and tumour M2-PK concentrations in healthy volunteers. Ann Clin Biochem, 2010; 47(3):259–263.

5. Nazario CED, Silva MR, Franco MS, Lanças FM. Evolution in miniaturized column liquid chromatography instrumentation and applications: An overview. J Chromatogr A. 2015; 1421:18–37.

6. Ferrige AG, Seddon MJ, Jarvis S, Skilling J, Aplin R. Maximum entropy deconvolution in electrospray mass spectrometry. Rapid Commun Mass Spectrom. 1991; 5(8):374–377.

7. O'Donnell LJ, Virjee J, Heaton KW. Detection of pseudodiarrhoea by simple clinical assessment of intestinal transit rate. BMJ. 1990; 300(6722):439–440.

8. Cohen SH, Gerding DN, Johnson S, et al. Clinical Practice Guidelines for *Clostridium difficile* Infection in Adults: 2010 Update by the Society for Healthcare Epidemiology of America (SHEA) and the Infectious Diseases Society of America (IDSA). Infect Control Hosp Epidemiol. 2010; 31(05):431–455.

9. Winter K, Xing L, Kassardjian A, Ward BJ. Vaccination against Clostridium difficile by Use of an Attenuated *Salmonella enterica* Serovar *Typhimurium* Vector (YS1646) Protects Mice from Lethal Challenge. Infect Immun. 2019; 87(8): :e00089-19.

10. Warren CA, Opstal E van, Ballard TE, et al. Amixicile, a Novel Inhibitor of Pyruvate:Ferredoxin Oxidoreductase, Shows Efficacy against *Clostridium difficile* in a Mouse Infection Model. Antimicrob Agents Chemother. 2012; 56(8):4103–4111.

S5. Supplemental Figures

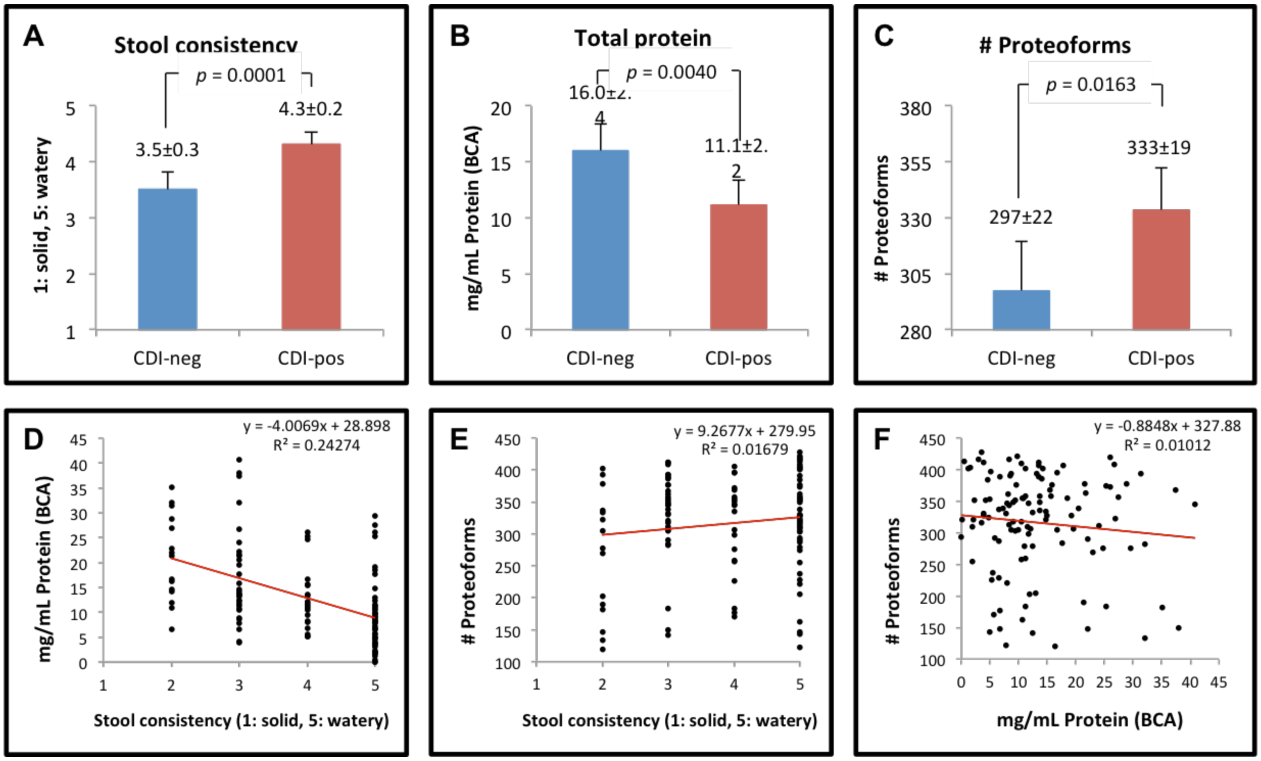

Figure S1. Meta-analysis of stool samples used for biomarker discovery of CDI in humans. **A–C:** Bar charts illustrating stool consistency, total protein concentration (mg/mL) and number of identified proteoforms in CDI-negative and CDI-positive samples. **D–F:** Linear regression plots demonstrating potential correlation between stool consistency, total protein concentration (mg/mL) and number of identified proteoforms in analyzed samples. Error bars represent 95% confidence interval. Significance levels were assessed by Student's *t*-test. Protein concentration was measured by the BCA protein assay.

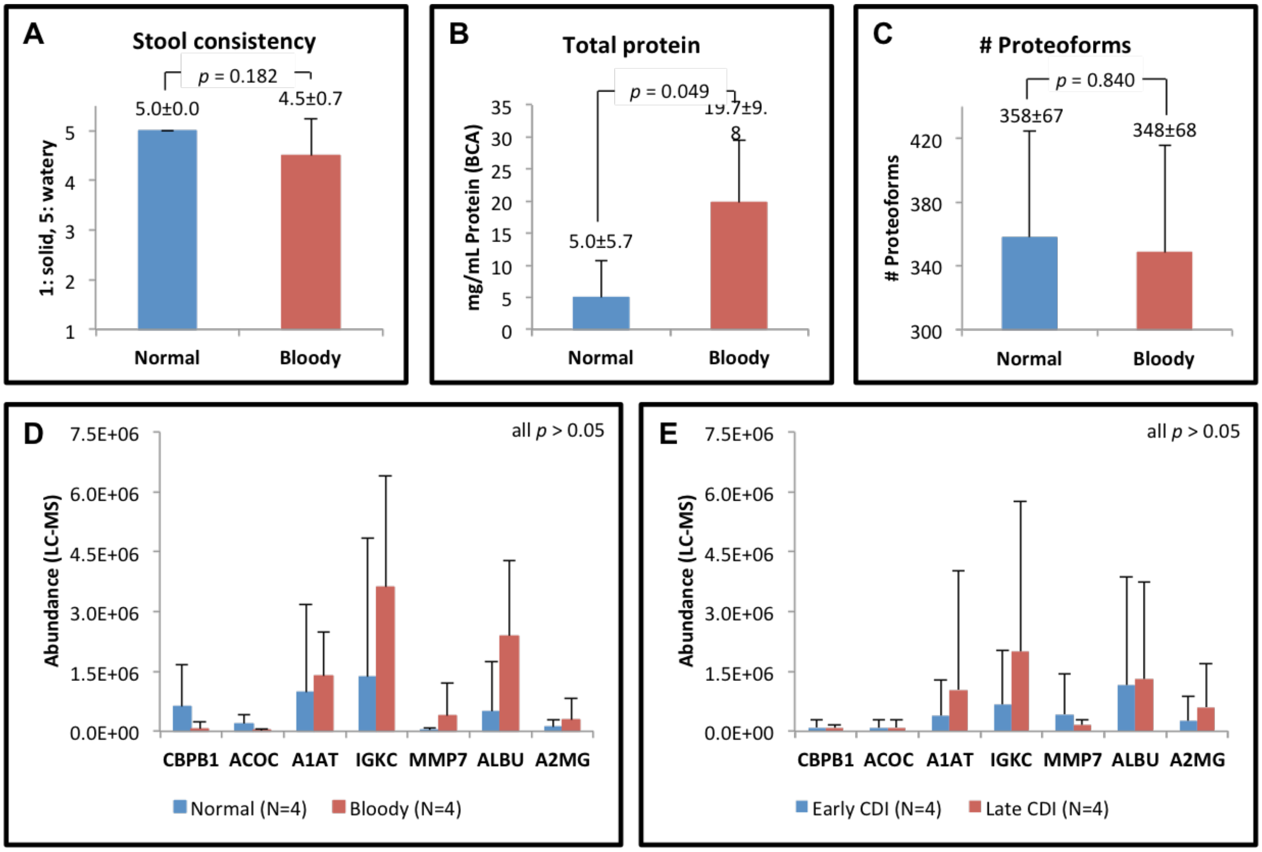

Figure S2. Potential effect of bleeding and disease progression on CDI stool properties. **A–D:** Bar charts illustrating stool consistency, total protein concentration (mg/mL), number of identified proteoforms and biomarker abundances (determined by LC-MS signal intensity) in patients with (N = 4) and without (N = 4) gastrointestinal bleeding. **E:** CDI biomarker abundances in four CDI patients at early-stage vs. late-stage CDI. Error bars represent 95% confidence interval. Significance levels were assessed by Student's *t*-test. Protein concentration was measured by the BCA protein assay.

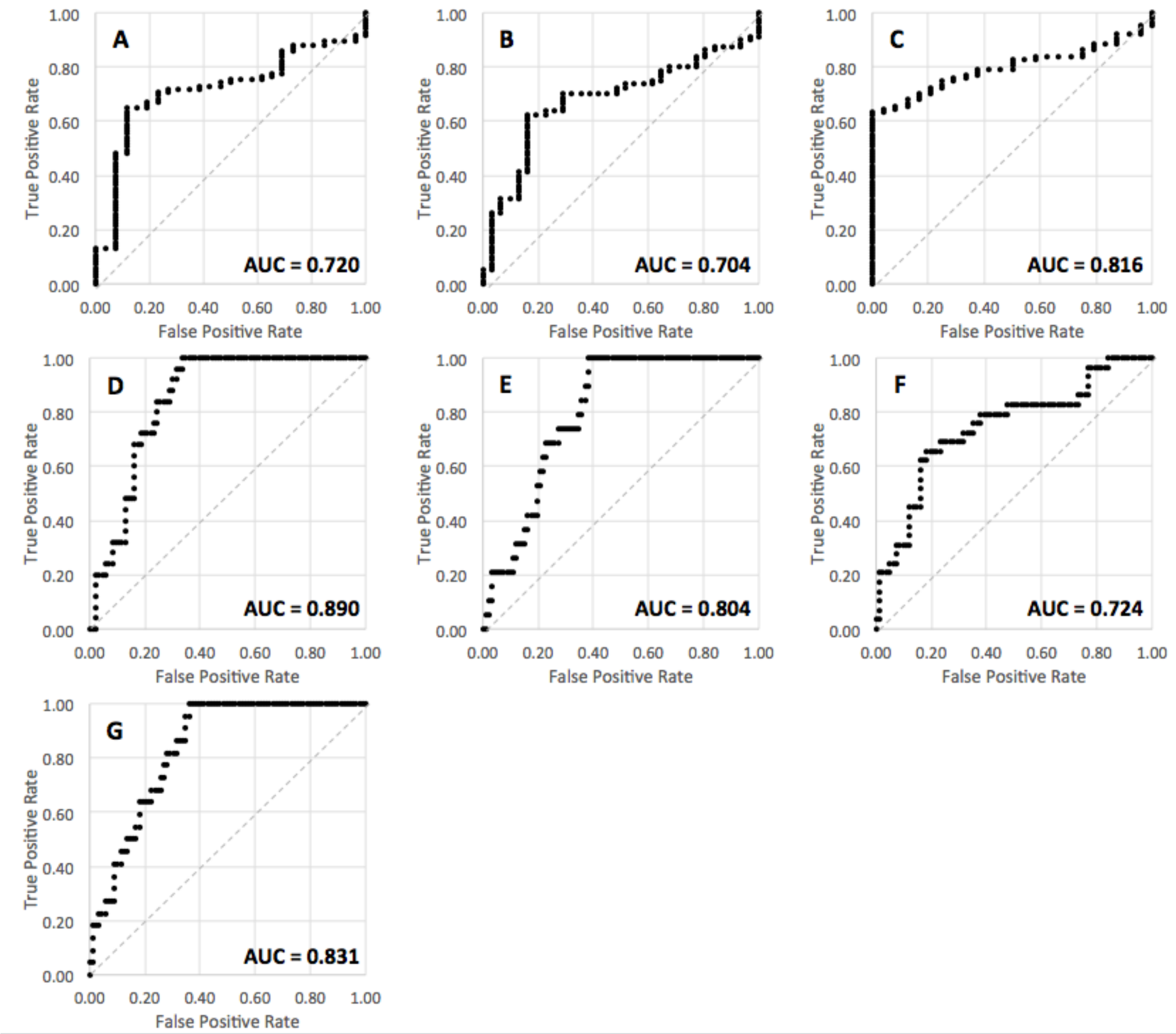

Figure S3. Receiver operating characteristic (ROC) plots for human fecal CDI biomarkers suggests that the identified biomarkers distinguish CDI-positive from CDI-negative samples with fair to good (AUC = 0.7–0.9) sensitivity and specificity. **A**: CBPB1; **B**: ACOC; **C**: A1AT; **D**: IGKC; **E**: MMP7; **F**: ALBU; **G**: A2MG.

(Next page):

Figure S4. Cerebral network in the context of subcellular localization illustrating known interactions between *Clostridioides difficile* infection biomarkers identified in humans (marked in red). ALB, A2M, SERPINA1, ACO1, MMP7, IGKC and CPB1 are each involved in 173, 137, 44, 14, 11, 1 and 1 interactions, respectively. Physical association and physical interaction are the most abundant interactions accounting for 248 and 103 interactions, respectively. A2M: alpha-2-macroglobulin; ACO1: cytoplasmic aconitate hydratase; ALB: serum albumin; CPB1: carboxypeptidase B; IGKC: immunoglobulin kappa constant; MMP7: matrix metalloproteinase 7; SERPINA1: alpha-1-antitrypsin.

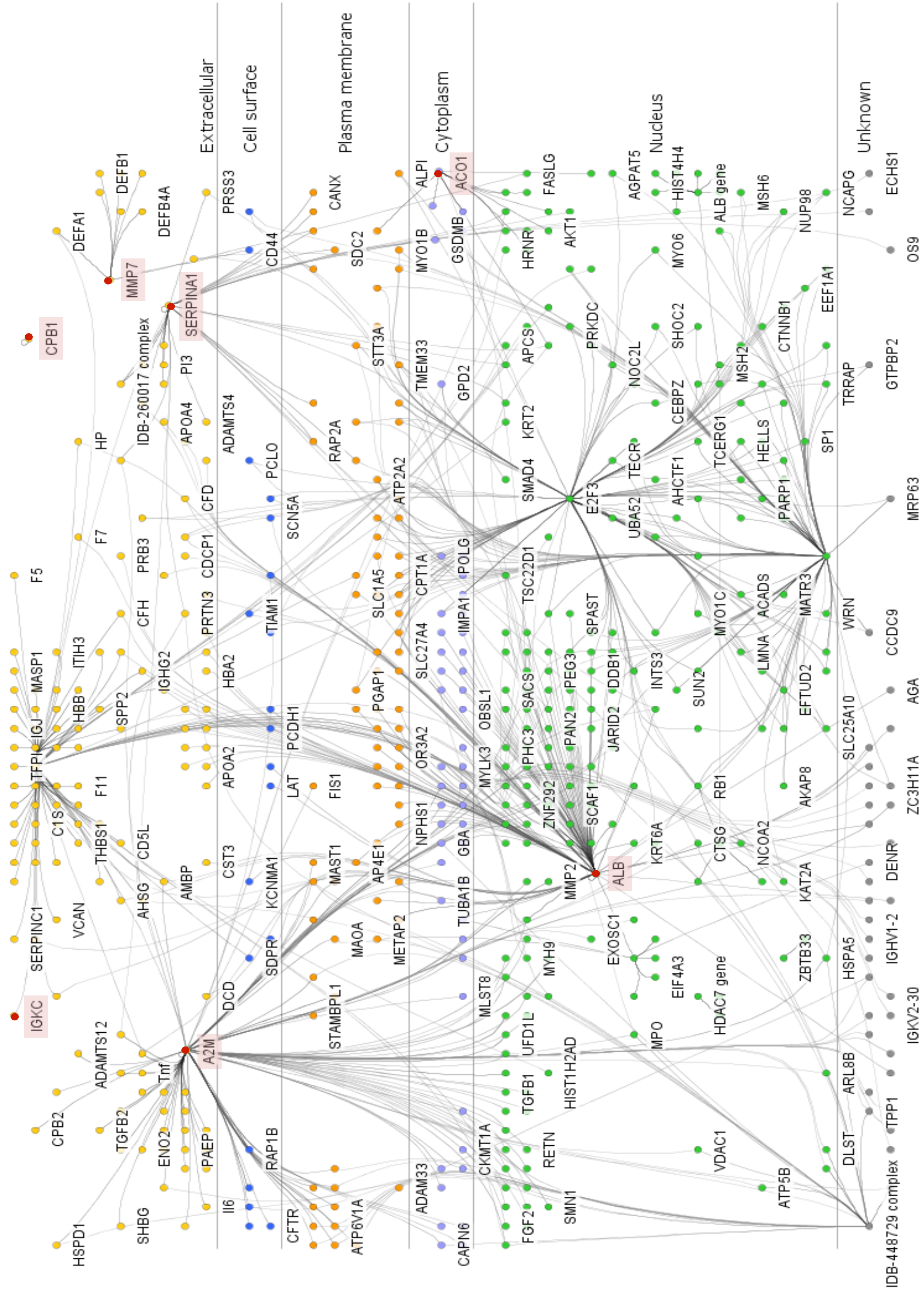
Extracellular
Cell surface
Plasma membrane
Cytoplasm
Nucleus
Unknown
IGKC
CPB1
MMP7
SERPINA1
A2M
AC01
ALB

S6. Supplemental Tables

Table S1. Pearson's coefficients of correlation between age, stool consistency (1 for solid to 5 for watery stool), protein content (BCA assay) and number of identified proteoforms in stool.

	**Age**	**Stool consistency**	**Stool protein content**	**# Proteoforms**
**Age**	**1.00**	-0.11	-0.03	0.11
**Stool consistency**	-0.11	**1.00**	-0.51	0.14
**Stool protein content**	-0.03	-0.51	**1.00**	-0.09
**# Proteoforms**	0.11	0.14	-0.09	**1.00**

Table S2. Pearson's coefficients of correlation between biomarker level, patient characteristics and selected clinical parameters. LC-MS signal intensity was used as a surrogate for biomarker level in stool. Moderate (|*r*| > 0.45) and strong (|*r*| > 0.90) correlations were marked in orange and red, respectively.

	**CBPB1**	**ACOC**	**A1AT**	**IGKC**	**MMP7**	**ALBU**	**A2MG**
**CBPB1**	**1.00**	0.46	-0.10	-0.24	-0.18	-0.24	-0.08
**ACOC**	0.46	**1.00**	0.03	-0.06	-0.01	-0.04	-0.03
**A1AT**	-0.10	0.03	**1.00**	0.25	0.01	0.19	-0.10
**IGKC**	-0.24	-0.06	0.25	**1.00**	0.50	**0.91**	0.28
**MMP7**	-0.18	-0.01	0.01	0.50	**1.00**	0.61	0.11
**ALBU**	-0.24	-0.04	0.19	**0.91**	0.61	**1.00**	0.27
**A2MG**	-0.08	-0.03	-0.10	0.28	0.11	0.27	**1.00**
**Age**	-0.05	0.11	-0.03	0.03	-0.05	0.02	-0.03
**Sex**	0.01	0.10	-0.06	-0.08	-0.01	-0.05	0.13
**Stool consistency**	0.00	0.08	0.22	0.24	0.08	0.26	-0.02
**Stool protein content**	-0.01	-0.09	-0.11	-0.19	-0.04	-0.18	-0.08
**WBC count**	-0.25	-0.16	0.01	0.06	0.07	0.16	-0.08
**Serum creatinine**	0.00	0.20	-0.03	-0.05	0.15	0.14	-0.11
**Serum albumin**	0.17	0.28	-0.08	0.18	0.07	-0.11	0.27
**Hypotension**	-0.15	-0.22	-0.03	-0.04	-0.08	0.06	0.35
**Shock**	-0.10	-0.15	0.12	-0.01	0.08	0.20	-0.09
**Ileus**	-0.07	-0.19	-0.03	-0.15	-0.05	-0.02	-0.06
**Megacolon**	-0.11	-0.16	0.05	-0.08	0.03	0.10	-0.10
**Antibiotic treatment**	0.15	-0.18	-0.15	-0.41	-0.30	-0.35	-0.13

Table S3. List of significantly ($p < 0.05$) over-represented pathways in *Clostridioides difficile* infected patients. *p*-Values were calculated by InnateDB analysis platform and corrected using the Benjamini-Hochberg correction method.

Pathway Name	Altered genes	Pathway genes	Pathway p-value (corrected)
Response to elevated platelet cytosolic Ca^{2+}	3	83	1.47E-05
Platelet degranulation	3	78	2.44E-05
HDL-mediated lipid transport	2	13	4.03E-05
Platelet activation, signaling and aggregation	3	219	1.10E-04
Lipoprotein metabolism	2	25	1.16E-04
Lipid digestion, mobilization, and transport	2	43	2.32E-04
Hemostasis	3	508	8.41E-04
Degradation of the extracellular matrix	2	116	1.13E-03
Extracellular matrix organization	2	266	5.28E-03
Metabolism of lipids and lipoproteins	2	554	2.00E-02
Metabolism	2	1535	1.22E-01

Table S4. List of significantly ($p < 0.05$) over-represented pathways in *Clostridioides difficile* infected mice. *p*-Values were calculated by InnateDB analysis platform and corrected using the Benjamini-Hochberg correction method.

Pathway Name	Altered genes	Pathway genes	Pathway p-value (corrected)
Degradation of the extracellular matrix	2	100	3.74E-03
Extracellular matrix organization	2	216	1.35E-02
Innate Immune System	2	450	4.57E-02
Immune System	2	884	1.32E-01
Metabolism	2	1414	2.50E-01

Table S5. List of protein-protein, protein-DNA and DNA-DNA interactions among the identified fecal human biomarkers of CDI (**Microsoft Excel file**).
