# Supplementary material for: Fecal host biomarkers predicting severity of Clostridioides difficile infection
Source: JCI Insight. 2021 Jan 11;6(1):e142976. doi: 10.1172/jci.insight.142976 (PMC7821589; doi:10.1172/jci.insight.142976)
Supplement: Supplemental Table 5 [file jciinsight-6-142976-s008.pdf]

Protein ID	Interaction Name	Species	Interaction Type	Interactor Type
P01009	LGALS3 interacts with SERPINA1	Homo sapiens	physical association	protein - protein
P01009	RB1 interacts with SERPINA1	Homo sapiens	physical association	protein - dna
P01009	NCOA2 interacts with SERPINA1	Homo sapiens	physical association	protein - dna
P01009	SERPINA1 interacts with ADAMTS4	Homo sapiens	physical association	protein - protein
P01009	HNF1A interacts with SERPINA1	Homo sapiens	physical association	protein - dna
P01009	ECHS1 physically interacts with SERPINA1	Homo sapiens	physical interaction	protein - protein
P01009	ZNF558 interacts with SERPINA1	Homo sapiens	physical association	protein - protein
P01009	RCHY1 physically interacts with SERPINA1	Homo sapiens	physical interaction	protein - protein
P01009	SDC2 physically interacts with SERPINA1	Homo sapiens	physical interaction	protein - protein
P01009	SERPINA1 interacts with VCP	Homo sapiens	physical association	protein - protein
P01009	SERPINA1 interacts with KLK3	Homo sapiens	physical association	protein - protein
P01009	SERPINA1 interacts with CANX	Homo sapiens	physical association	protein - protein
P01009	PRSS3 interacts with SERPINA1	Homo sapiens	physical association	protein - protein
P01009	SERPINA1 physically interacts with PRTN3	Homo sapiens	physical interaction	protein - protein
P01009	PI3 physically interacts with SERPINA1	Homo sapiens	physical interaction	protein - protein
P01009	SERPINA1 physically interacts with LOC780933	Homo sapiens/Bos taurus	physical interaction	protein - protein
P01009	SERPINA1 interacts with MIS12	Homo sapiens	physical association	protein - protein
P01009	SERPINA1 physically interacts with KLK13	Homo sapiens	physical interaction	protein - protein
P01009	SERPINA1 interacts with OS9	Homo sapiens	physical association	protein - protein
P01009	GZMM interacts with SERPINA1	Homo sapiens	physical association	protein - protein
P01009	Cleavage reaction involving GZMM and SERPINA1	Homo sapiens	protein cleavage	protein - protein
P01009	SSR1 interacts with SERPINA1	Homo sapiens	physical association	protein - protein
P01009	HNF4A interacts with SERPINA1	Homo sapiens	association	protein - dna
P01009	GSDMB physically interacts with SERPINA1	Homo sapiens	physical interaction	protein - protein
P01009	ATP5C1 physically interacts with SERPINA1	Homo sapiens	physical interaction	protein - protein
P01009	E2F4 interacts with SERPINA1	Homo sapiens	physical association	protein - dna
P01009	FN1 interacts with SERPINA1	Homo sapiens	physical association	protein - protein
P01009	SERPINA1 physically interacts with RAP2A	Homo sapiens	physical interaction	protein - protein
P01009	DERL1 interacts with SERPINA1	Homo sapiens	physical association	protein - protein
P01009	SERPINA1 physically interacts with LOC100848132	Homo sapiens/Bos taurus	physical interaction	protein - protein
P01009	SERPINA1 physically associates with SERPINA1	Homo sapiens	physical association	protein - protein
P01009	CTSG interacts with SERPINA1	Homo sapiens	physical association	protein - protein
P01009	HNF4A interacts with SERPINA1	Homo sapiens	physical association	protein - dna
P01009	ERLEC1 interacts with SERPINA1	Homo sapiens	physical association	protein - protein
P01009	SERPINA1 interacts with DERL2	Homo sapiens	physical association	protein - protein
P01009	SERPINA1 physically interacts with SMAD4	Homo sapiens	physical interaction	protein - protein
P01009	SERPINA1 physically interacts with VKORC1	Homo sapiens	physical interaction	protein - protein
P01009	UBC interacts with SERPINA1	Homo sapiens	physical association	protein - protein
P01009	Complex of 3 interactors	Homo sapiens/Bos taurus	physical association	protein - protein
P01009	Cleavage reaction involving LOC780933 and SERPINA1	Bos taurus/Homo sapiens	cleavage	protein - protein
P01009	SERPINA1 interacts with LRP1	Homo sapiens	physical association	protein - protein
P01009	SERPINA1 interacts with HNF1A	Homo sapiens	physical association	protein - dna
P01009	SERPINA1 interacts with HNF4A	Homo sapiens	physical association	protein - dna
P01009	ELANE interacts with SERPINA1	Homo sapiens	physical association	protein - protein
P01023	IGSF8 physically interacts with A2M	Homo sapiens	physical interaction	protein - protein
P01023	RNF32 physically interacts with A2M	Homo sapiens	physical interaction	protein - protein
P01023	LGALS3 interacts with A2M	Homo sapiens	physical association	protein - protein
P01023	H2AFX interacts with A2M	Homo sapiens	physical association	protein - protein
P01023	EXOSC1 physically interacts with A2M	Homo sapiens	physical interaction	protein - protein
P01023	FIS1 physically interacts with A2M	Homo sapiens	physical interaction	protein - protein
P01023	A2M physically interacts with SEPT8	Homo sapiens	physical interaction	protein - protein
P01023	EGLN2 physically interacts with A2M	Homo sapiens	physical interaction	protein - protein
P01023	LCAT physically interacts with A2M	Homo sapiens	physical interaction	protein - protein
P01023	A2M interacts with CFTR	Homo sapiens	physical association	protein - protein

P01023	SMN1 interacts with A2M	Homo sapiens	physical association	protein - protein
P01023	MLST8 physically interacts with A2M	Homo sapiens	physical interaction	protein - protein
P01023	NOS3 physically interacts with A2M	Homo sapiens	physical interaction	protein - protein
P01023	A2M interacts with MMP2	Homo sapiens	physical association	protein - protein
P01023	CDC37 physically interacts with A2M	Homo sapiens	physical interaction	protein - protein
P01023	A2M physically interacts with H2AFX	Homo sapiens	physical interaction	protein - protein
P01023	A2M interacts with TP63	Homo sapiens	physical association	protein - protein
P01023	NCDN physically interacts with A2M	Homo sapiens	physical interaction	protein - protein
P01023	LCAT interacts with A2M	Homo sapiens	physical association	protein - protein
P01023	ECSIT physically interacts with A2M	Homo sapiens	physical interaction	protein - protein
P01023	APP physically interacts with A2M	Homo sapiens	physical interaction	protein - protein
P01023	PRDX2 physically interacts with A2M	Homo sapiens	physical interaction	protein - protein
P01023	A2M physically interacts with GEM	Homo sapiens	physical interaction	protein - protein
P01023	IL1B interacts with A2M	Homo sapiens	physical association	protein - protein
P01023	IL10 physically interacts with A2M	Homo sapiens	physical interaction	protein - protein
P01023	ADAMTS1 interacts with A2M	Homo sapiens	physical association	protein - protein
P01023	A2M interacts with GZMA	Homo sapiens	physical association	protein - protein
P01023	PAEP physically interacts with A2M	Homo sapiens	physical interaction	protein - protein
P01023	A2M interacts with KLK3	Homo sapiens	physical association	protein - protein
P01023	ELAVL3 physically interacts with A2M	Homo sapiens	physical interaction	protein - protein
P01023	CYP2C8 physically interacts with A2M	Homo sapiens	physical interaction	protein - protein
P01023	ATP1A1 physically interacts with A2M	Homo sapiens	physical interaction	protein - protein
P01023	A2M interacts with LGALS8	Homo sapiens	physical association	protein - protein
P01023	A2M interacts with NUDT21	Homo sapiens	physical association	protein - protein
P01023	Cleavage reaction involving ADAMTS7 and A2M	Homo sapiens	protein cleavage	protein - protein
P01023	Cleavage reaction involving ADAMTS12 and A2M	Homo sapiens	protein cleavage	protein - protein
P01023	A2M interacts with LGALS9	Homo sapiens	physical association	protein - protein
P01023	A2M physically interacts with RAP1B	Homo sapiens	physical interaction	protein - protein
P01023	FBXW4 physically interacts with A2M	Homo sapiens	physical interaction	protein - protein
P01023	A2M physically interacts with CDC42	Homo sapiens	physical interaction	protein - protein
P01023	TTR interacts with A2M	Homo sapiens	physical association	protein - protein
P01023	A2M physically interacts with HSPA5	Homo sapiens	physical interaction	protein - protein
P01023	A2M interacts with APP	Homo sapiens	physical association	protein - protein
P01023	APOE physically interacts with A2M	Homo sapiens	physical interaction	protein - protein
P01023	Colocalization of A2M and ACTB :: SLC25A5 :: SLC25A6 :: MA	Homo sapiens	colocalization	protein - protein
P01023	A2M physically interacts with CDK2AP2	Homo sapiens	physical interaction	protein - protein
P01023	A2M physically interacts with TSC22D1	Homo sapiens	physical interaction	protein - protein
P01023	A2M physically interacts with TTR	Homo sapiens	physical interaction	protein - protein
P01023	IL4 physically interacts with A2M	Homo sapiens	physical interaction	protein - protein
P01023	NGF physically interacts with A2M	Homo sapiens	physical interaction	protein - protein
P01023	CYP2C18 physically interacts with A2M	Homo sapiens	physical interaction	protein - protein
P01023	A2M interacts with TNFRSF14	Homo sapiens	physical association	protein - protein
P01023	PRAM1 physically interacts with A2M	Homo sapiens	physical interaction	protein - protein
P01023	MGEA5 physically interacts with A2M	Homo sapiens	physical interaction	protein - protein
P01023	RAB3A physically interacts with A2M	Homo sapiens	physical interaction	protein - protein
P01023	A2M interacts with TGIF1	Homo sapiens	physical association	protein - protein
P01023	SHBG physically interacts with A2M	Homo sapiens	physical interaction	protein - protein
P01023	FBXL12 physically interacts with A2M	Homo sapiens	physical interaction	protein - protein
P01023	A2M interacts with TK1	Homo sapiens	physical association	protein - protein
P01023	LONP1 physically interacts with A2M	Homo sapiens	physical interaction	protein - protein
P01023	ELANE interacts with A2M	Homo sapiens	physical association	protein - protein
P01023	A2M physically interacts with TNFRSF14	Homo sapiens	physical interaction	protein - protein
P01023	A2M physically interacts with MMP2	Homo sapiens	physical interaction	protein - protein
P01023	STAMBPL1 physically interacts with A2M	Homo sapiens	physical interaction	protein - protein
P01023	A2M physically interacts with CDKN1A	Homo sapiens	physical interaction	protein - protein

P01023	SWSAP1 physically interacts with A2M	Homo sapiens	physical interaction	protein - protein
P01023	A2M interacts with AMBP	Homo sapiens	physical association	protein - protein
P01023	HIPK1 interacts with A2M	Homo sapiens	physical association	protein - protein
P01023	PAXIP1 physically interacts with A2M	Homo sapiens	physical interaction	protein - protein
P01023	RAP1B interacts with A2M	Homo sapiens	physical association	protein - protein
P01023	A2M physically interacts with KLK13	Homo sapiens	physical interaction	protein - protein
P01023	CDK7 interacts with A2M	Homo sapiens	physical association	protein - protein
P01023	A2M physically interacts with MAST1	Homo sapiens	physical interaction	protein - protein
P01023	MAPK6 interacts with A2M	Homo sapiens	physical association	protein - protein
P01023	C11orf58 interacts with A2M	Homo sapiens	physical association	protein - protein
P01023	A2M physically interacts with HMOX2	Homo sapiens	physical interaction	protein - protein
P01023	A2M physically interacts with A2M	Homo sapiens	physical interaction	protein - protein
P01023	CDK11A interacts with A2M	Homo sapiens	physical association	protein - protein
P01023	A2M physically interacts with TK1	Homo sapiens	physical interaction	protein - protein
P01023	ENO2 physically interacts with A2M	Homo sapiens	physical interaction	protein - protein
P01023	A2M interacts with TSC22D1	Homo sapiens	physical association	protein - protein
P01023	AP1M2 physically interacts with A2M	Homo sapiens	physical interaction	protein - protein
P01023	A2M physically interacts with TGM2	Homo sapiens	physical interaction	protein - protein
P01023	TRAPPC5 physically interacts with A2M	Homo sapiens	physical interaction	protein - protein
P01023	MYOC physically interacts with A2M	Homo sapiens	physical interaction	protein - protein
P01023	A2M physically interacts with RPP14	Homo sapiens	physical interaction	protein - protein
P01023	Complex of 3 interactors	Homo sapiens	cleavage reaction	protein - protein
P01023	IFIT5 physically interacts with A2M	Homo sapiens	physical interaction	protein - protein
P01023	ACTB physically interacts with A2M	Homo sapiens	physical interaction	protein - protein
P01023	A2M interacts with HMOX2	Homo sapiens	physical association	protein - protein
P01023	GCDH physically interacts with A2M	Homo sapiens	physical interaction	protein - protein
P01023	A2M physically interacts with CPB2	Homo sapiens	physical interaction	protein - protein
P01023	IFIT3 physically interacts with A2M	Homo sapiens	physical interaction	protein - protein
P01023	SEPT8 interacts with A2M	Homo sapiens	physical association	protein - protein
P01023	RETN physically interacts with A2M	Homo sapiens	physical interaction	protein - protein
P01023	APOE interacts with A2M	Homo sapiens	physical association	protein - protein
P01023	TYRO3 physically interacts with A2M	Homo sapiens	physical interaction	protein - protein
P01023	A2M physically interacts with NUDT21	Homo sapiens	physical interaction	protein - protein
P01023	FARSA physically interacts with A2M	Homo sapiens	physical interaction	protein - protein
P01023	A2M physically interacts with SMN1	Homo sapiens	physical interaction	protein - protein
P01023	LEP physically interacts with A2M	Homo sapiens	physical interaction	protein - protein
P01023	EVI5L physically interacts with A2M	Homo sapiens	physical interaction	protein - protein
P01023	A2M interacts with IL8	Homo sapiens	physical association	protein - protein
P01023	A2M interacts with CDKN1A	Homo sapiens	physical association	protein - protein
P01023	A2M interacts with IL10	Homo sapiens	physical association	protein - protein
P01023	RHEB physically interacts with A2M	Homo sapiens	physical interaction	protein - protein
P01023	RAD23A physically interacts with A2M	Homo sapiens	physical interaction	protein - protein
P01023	A2M physically interacts with B2M	Homo sapiens	physical interaction	protein - protein
P01023	A2M physically interacts with TGIF1	Homo sapiens	physical interaction	protein - protein
P01023	UMPS physically interacts with A2M	Homo sapiens	physical interaction	protein - protein
P01023	DNAJB1 physically interacts with A2M	Homo sapiens	physical interaction	protein - protein
P01023	A2M interacts with IL2	Homo sapiens	physical association	protein - protein
P01023	IL6 interacts with A2M	Homo sapiens	physical association	protein - protein
P01023	A2M interacts with TNF	Homo sapiens	physical association	protein - protein
P01023	A2M interacts with ANXA7	Homo sapiens	physical association	protein - protein
P01023	A2M interacts with TGFB2	Homo sapiens	physical association	protein - protein
P01023	PDGFB interacts with A2M	Homo sapiens	physical association	protein - protein
P01023	PAEP interacts with A2M	Homo sapiens	association	protein - protein
P01023	A2M interacts with TGFB1	Homo sapiens	physical association	protein - protein
P01023	A2M physically interacts with GRB2	Homo sapiens	physical interaction	protein - protein

P01023	A2M interacts with FGF2	Homo sapiens	physical association	protein - protein
P01023	A2M interacts with NGF	Homo sapiens	physical association	protein - protein
P01023	ATF7IP interacts with A2M	Homo sapiens	physical association	protein - protein
P01023	A2M physically interacts with C11orf58	Homo sapiens	physical interaction	protein - protein
P01023	A2M physically interacts with UFD1L	Homo sapiens	physical interaction	protein - protein
P01023	A2M physically interacts with ANXA7	Homo sapiens	physical interaction	protein - protein
P01023	A2M interacts with CDK2AP2	Homo sapiens	physical association	protein - protein
P01023	TUBA1B physically interacts with A2M	Homo sapiens	physical interaction	protein - protein
P01023	A2M interacts with RPP14	Homo sapiens	physical association	protein - protein
P01023	A2M interacts with LRP1	Homo sapiens	association	protein - protein
P01023	A2M interacts with UFD1L	Homo sapiens	physical association	protein - protein
P01023	Complex of 34 interactors	Homo sapiens	colocalization	protein - protein
P01023	A2M interacts with IL4	Homo sapiens	physical association	protein - protein
P01023	A2M interacts with Il18	Homo sapiens/Mus musculus	physical association	protein - protein
P01023	A2M interacts with Il6	Homo sapiens/Mus musculus	physical association	protein - protein
P01023	A2M interacts with Tnf	Homo sapiens/Mus musculus	physical association	protein - protein
P01023	FBXO6 interacts with A2M	Homo sapiens	physical association	protein - protein
P02768	OBSL1 interacts with ALB	Homo sapiens	physical association	protein - protein
P02768	CROT interacts with ALB	Homo sapiens	physical association	protein - protein
P02768	PRSS3 interacts with ALB	Homo sapiens	physical association	protein - protein
P02768	HBA2 interacts with ALB	Homo sapiens	physical association	protein - protein
P02768	ALB physically interacts with NR5A2	Homo sapiens	physical interaction	protein - protein
P02768	PLAG1 interacts with ALB	Homo sapiens	physical association	protein - protein
P02768	SPAST interacts with ALB	Homo sapiens	physical association	protein - protein
P02768	Complex of 247 interactors	Homo sapiens	association	protein - protein
P02768	KRT14 interacts with ALB	Homo sapiens	physical association	protein - protein
P02768	APOA1 interacts with ALB	Homo sapiens	physical association	protein - protein
P02768	DERL1 interacts with ALB	Homo sapiens	physical association	protein - protein
P02768	RLF interacts with ALB	Homo sapiens	physical association	protein - protein
P02768	AP4E1 interacts with ALB	Homo sapiens	physical association	protein - protein
P02768	PARK2 interacts with ALB	Homo sapiens	physical association	protein - protein
P02768	ALB physically associates with ALB	Homo sapiens	physical association	protein - protein
P02768	ITGA2 interacts with ALB	Homo sapiens	physical association	protein - protein
P02768	PPBP interacts with ALB	Homo sapiens	physical association	protein - protein
P02768	KRT16 interacts with ALB	Homo sapiens	physical association	protein - protein
P02768	RYR2 interacts with ALB	Homo sapiens	physical association	protein - protein
P02768	SLC25A13 interacts with ALB	Homo sapiens	physical association	protein - protein
P02768	RANBP3 interacts with ALB	Homo sapiens	physical association	protein - protein
P02768	EXOC6 interacts with ALB	Homo sapiens	physical association	protein - protein
P02768	CEP44 interacts with ALB	Homo sapiens	physical association	protein - protein
P02768	CLCA2 interacts with ALB	Homo sapiens	physical association	protein - protein
P02768	FN1 interacts with ALB	Homo sapiens	physical association	protein - protein
P02768	IGHG2 interacts with ALB	Homo sapiens	physical association	protein - protein
P02768	LAT interacts with ALB	Homo sapiens	physical association	protein - protein
P02768	Complex of 103 interactors	Homo sapiens	physical association	protein - protein
P02768	HP interacts with ALB	Homo sapiens	physical association	protein - protein
P02768	APOA2 interacts with ALB	Homo sapiens	physical association	protein - protein
P02768	OR3A2 interacts with ALB	Homo sapiens	physical association	protein - protein
P02768	KAT2B interacts with ALB	Homo sapiens	physical association	protein - dna
P02768	ELP3 interacts with ALB	Homo sapiens	physical association	protein - dna
P02768	CRB1 interacts with ALB	Homo sapiens	physical association	protein - protein
P02768	AMPD3 interacts with ALB	Homo sapiens	physical association	protein - protein
P02768	PLA2G4F interacts with ALB	Homo sapiens	physical association	protein - protein
P02768	CAMTA1 interacts with ALB	Homo sapiens	physical association	protein - protein
P02768	QTRTD1 interacts with ALB	Homo sapiens	physical association	protein - protein

P02768	FAM71E2 interacts with ALB	Homo sapiens	physical association	protein - protein
P02768	JARID2 interacts with ALB	Homo sapiens	physical association	protein - protein
P02768	H3F3A interacts with ALB	Homo sapiens	physical association	protein - dna
P02768	SMARCA5 interacts with ALB	Homo sapiens	physical association	protein - dna
P02768	ALB interacts with HNF1A	Homo sapiens	physical association	protein - dna
P02768	ZNF558 interacts with ALB	Homo sapiens	physical association	protein - protein
P02768	SMARCA4 interacts with ALB	Homo sapiens	physical association	protein - dna
P02768	GSN interacts with ALB	Homo sapiens	physical association	protein - protein
P02768	DGKG interacts with ALB	Homo sapiens	physical association	protein - protein
P02768	TTN interacts with ALB	Homo sapiens	physical association	protein - protein
P02768	CDC45 interacts with ALB	Homo sapiens	physical association	protein - protein
P02768	GJC2 interacts with ALB	Homo sapiens	physical association	protein - protein
P02768	AGA interacts with ALB	Homo sapiens	physical association	protein - protein
P02768	OR2T6 interacts with ALB	Homo sapiens	physical association	protein - protein
P02768	ALB interacts with CEBPB	Homo sapiens	physical association	protein - dna
P02768	SLA2 interacts with ALB	Homo sapiens	physical association	protein - protein
P02768	ALB interacts with UBD	Homo sapiens	physical association	protein - protein
P02768	SGOL2 interacts with ALB	Homo sapiens	physical association	protein - protein
P02768	MYL4 interacts with ALB	Homo sapiens	physical association	protein - protein
P02768	IL7R interacts with ALB	Homo sapiens	physical association	protein - protein
P02768	ALB interacts with USP37	Homo sapiens	physical association	protein - protein
P02768	AHSG interacts with ALB	Homo sapiens	physical association	protein - protein
P02768	HIST4H4 interacts with ALB	Homo sapiens	physical association	protein - dna
P02768	C4A interacts with ALB	Homo sapiens	physical association	protein - protein
P02768	PCDH1 interacts with ALB	Homo sapiens	physical association	protein - protein
P02768	ITGB5 interacts with ALB	Homo sapiens	physical association	protein - protein
P02768	ALB physically interacts with IMMT	Homo sapiens	physical interaction	protein - protein
P02768	IGHG1 interacts with ALB	Homo sapiens	physical association	protein - protein
P02768	THRAP3 interacts with ALB	Homo sapiens	physical association	protein - protein
P02768	APOE interacts with ALB	Homo sapiens	physical association	protein - protein
P02768	APOC1 interacts with ALB	Homo sapiens	physical association	protein - protein
P02768	KRT6B interacts with ALB	Homo sapiens	physical association	protein - protein
P02768	ZNF292 interacts with ALB	Homo sapiens	physical association	protein - protein
P02768	CFD interacts with ALB	Homo sapiens	physical association	protein - protein
P02768	DCD interacts with ALB	Homo sapiens	physical association	protein - protein
P02768	DICER1 interacts with ALB	Homo sapiens	physical association	protein - protein
P02768	APOC3 interacts with ALB	Homo sapiens	physical association	protein - protein
P02768	KRT1 interacts with ALB	Homo sapiens	physical association	protein - protein
P02768	ALB interacts with YWHAG	Homo sapiens	physical association	protein - protein
P02768	CNOT1 interacts with ALB	Homo sapiens	physical association	protein - protein
P02768	GCN1L1 interacts with ALB	Homo sapiens	physical association	protein - protein
P02768	ALB interacts with TK1	Homo sapiens	physical association	protein - protein
P02768	PAN2 interacts with ALB	Homo sapiens	physical association	protein - protein
P02768	KLK3 interacts with ALB	Homo sapiens	physical association	protein - protein
P02768	ALB interacts with IQCB1	Homo sapiens	physical association	protein - protein
P02768	OR8D2 interacts with ALB	Homo sapiens	physical association	protein - protein
P02768	TIAM1 interacts with ALB	Homo sapiens	physical association	protein - protein
P02768	SCAF1 interacts with ALB	Homo sapiens	physical association	protein - protein
P02768	TTPAL interacts with ALB	Homo sapiens	physical association	protein - protein
P02768	APOC4 interacts with ALB	Homo sapiens	physical association	protein - protein
P02768	KCNMA1 interacts with ALB	Homo sapiens	physical association	protein - protein
P02768	BBC3 interacts with ALB	Homo sapiens	physical association	protein - protein
P02768	DDB1 interacts with ALB	Homo sapiens	physical association	protein - protein
P02768	SETX interacts with ALB	Homo sapiens	physical association	protein - protein
P02768	PFDN1 interacts with ALB	Homo sapiens	physical association	protein - protein

P02768	KRT10 interacts with ALB	Homo sapiens	physical association	protein - protein
P02768	CFH interacts with ALB	Homo sapiens	physical association	protein - protein
P02768	FCGRT physically interacts with ALB	Homo sapiens	physical interaction	protein - protein
P02768	MYLK3 interacts with ALB	Homo sapiens	physical association	protein - protein
P02768	IGDCC4 interacts with ALB	Homo sapiens	physical association	protein - protein
P02768	APP physically interacts with ALB	Homo sapiens	physical interaction	protein - protein
P02768	CDCP1 interacts with ALB	Homo sapiens	physical association	protein - protein
P02768	TFRC physically interacts with ALB	Homo sapiens	physical interaction	protein - protein
P02768	ALB physically associates with ALB	Homo sapiens	physical association	protein - protein
P02768	ST13 interacts with ALB	Homo sapiens	physical association	protein - protein
P02768	ADRA1B interacts with ALB	Homo sapiens	physical association	protein - protein
P02768	CABLES1 interacts with ALB	Homo sapiens	physical association	protein - protein
P02768	ALB physically interacts with TK1	Homo sapiens	physical interaction	protein - protein
P02768	APOA4 interacts with ALB	Homo sapiens	physical association	protein - protein
P02768	ITIH1 interacts with ALB	Homo sapiens	physical association	protein - protein
P02768	LDB3 interacts with ALB	Homo sapiens	physical association	protein - protein
P02768	NPHS1 interacts with ALB	Homo sapiens	physical association	protein - protein
P02768	TTR interacts with ALB	Homo sapiens	physical association	protein - protein
P02768	PF4V1 interacts with ALB	Homo sapiens	physical association	protein - protein
P02768	GABBR1 interacts with ALB	Homo sapiens	physical association	protein - protein
P02768	SLC9A8 interacts with ALB	Homo sapiens	physical association	protein - protein
P02768	PDE4B interacts with ALB	Homo sapiens	physical association	protein - protein
P02768	F2 interacts with ALB	Homo sapiens	physical association	protein - protein
P02768	CTSL1 interacts with ALB	Homo sapiens	physical association	protein - protein
P02768	CHKB interacts with ALB	Homo sapiens	physical association	protein - protein
P02768	ALB interacts with UBC	Homo sapiens	physical association	protein - protein
P02768	HPX interacts with ALB	Homo sapiens	physical association	protein - protein
P02768	ALB physically interacts with PFDN1	Homo sapiens	physical interaction	protein - protein
P02768	E2F3 interacts with RFC1 :: RHOT1 :: RHOT2 :: PCLO :: INTS5 :: GPR116 :: GPAM :: GPD2 :: SLC16A3 :: LBR :: TCF7L2 :: CLASP1 :: CEBPZ :: KAT2A :: SLC25A5 :: DHX33 :: RAB13 :: ATP2A2 :: SLC25A3 :: NOC2L :: TECR :: L3MBTL3 :: ZNF300 :: ZC3H11A :: NUP98 :: MYBBP1A :: XRCC5 :: RPS3 :: SEC61A1 :: AGPAT5 :: MYO6 :: IMPA1 :: NUP205 :: INTS3 :: NUMA1 :: TCERG1 :: SCD :: PRKDC :: EEF1A1 :: CTNNB1 :: MSH6 :: MRP63 :: LMNA :: NCAPG :: SHOC2 :: KDM1A :: DHX9 :: ENOX2 :: NAT10 :: RB1 :: CTNNA1 :: NCL :: HADHA :: PMM1 :: ZFYVE20 :: ALB :: MYO1B :: INTS6 :: ATP1A1 :: EFTUD2 :: TRIM28 :: HLTF :: CPT1A :: SUN2 :: SFMBT1 :: KRT14 :: MSH2 :: HSPA6 :: DDX21 :: PARP1 :: HSPA9 :: GTPBP2 :: AFG3L2 :: HSPA5 :: TNXB :: SLC25A4 :: XRCC6 :: NOTCH3 :: HELLS :: CCDC9 :: TRRAP :: STT3A :: ZNF148 :: LRPPRC :: SLC27A4 :: SMPD4 :: ATAD3A :: SLC25A6 :: SLC2A1 :: DHCR7 :: IMMT :: TFRC :: PGAP1 :: MATR3 :: DDB1 :: TRIM23 :: KRT1 :: ZBED4 :: ACADS :: HNRNPU :: UBA52 :: AHCTF1 :: NUP93 :: ALPI :: SMARCA1 :: AKAP8 :: POLG :: SP1 :: WRN	Homo sapiens	association	protein - protein
P02768	PRB3 physically interacts with ALB	Homo sapiens	physical interaction	protein - protein
P02768	NLRC4 interacts with ALB	Homo sapiens	physical association	protein - protein
P02768	GFAP interacts with ALB	Homo sapiens	physical association	protein - protein
P02768	SPATA31A7 interacts with ALB	Homo sapiens	physical association	protein - protein
P02768	Complex of 8 interactors	Homo sapiens	association	protein - protein
P02768	ALB interacts with AMBP	Homo sapiens	physical association	protein - protein
P02768	AP1M1 interacts with ALB	Homo sapiens	physical association	protein - protein
P02768	TLN2 interacts with ALB	Homo sapiens	physical association	protein - protein

P02768	ZNF232 interacts with ALB	Homo sapiens	physical association	protein - protein
P02768	LUC7L2 physically interacts with ALB	Homo sapiens	physical interaction	protein - protein
P02768	ALB interacts with PSMA3	Homo sapiens	physical association	protein - protein
P02768	FBXO25 interacts with ALB	Homo sapiens	physical association	protein - protein
P02768	SACS interacts with ALB	Homo sapiens	physical association	protein - protein
P02768	ALB interacts with PSMD4	Homo sapiens	physical association	protein - protein
P02768	F7 interacts with ALB	Homo sapiens	physical association	protein - protein
P02768	KIAA0232 interacts with ALB	Homo sapiens	physical association	protein - protein
P02768	NCOA3 interacts with ALB	Homo sapiens	physical association	protein - protein
P02768	ALB interacts with DMWD	Homo sapiens	physical association	protein - protein
P02768	KRT5 interacts with ALB	Homo sapiens	physical association	protein - protein
P02768	RANBP2 interacts with ALB	Homo sapiens	physical association	protein - protein
P02768	ALB interacts with EEF2K	Homo sapiens	physical association	protein - protein
P02768	CTAGE5 interacts with ALB	Homo sapiens	physical association	protein - protein
P02768	SH3BP5 interacts with ALB	Homo sapiens	physical association	protein - protein
P02768	ALB interacts with UIMC1	Homo sapiens	physical association	protein - protein
P02768	DMD interacts with ALB	Homo sapiens	physical association	protein - protein
P02768	PDZRN4 interacts with ALB	Homo sapiens	physical association	protein - protein
P02768	PEG3 interacts with ALB	Homo sapiens	physical association	protein - protein
P02768	ATM interacts with ALB	Homo sapiens	physical association	protein - protein
P02768	ALB interacts with IMMT	Homo sapiens	physical association	protein - protein
P02768	PRSS1 interacts with ALB	Homo sapiens	physical association	protein - protein
P02768	ETF1 interacts with ALB	Homo sapiens	physical association	protein - protein
P02768	TRAPPC11 interacts with ALB	Homo sapiens	physical association	protein - protein
P02768	SCN5A interacts with ALB	Homo sapiens	physical association	protein - protein
P02768	GRAP2 interacts with ALB	Homo sapiens	physical association	protein - protein
P02768	PHC3 interacts with ALB	Homo sapiens	physical association	protein - protein
P02768	PALB2 interacts with ALB	Homo sapiens	physical association	protein - protein
P02768	SORBS3 interacts with ALB	Homo sapiens	physical association	protein - protein
P02768	SERPING1 interacts with ALB	Homo sapiens	physical association	protein - protein
P02768	FUS interacts with ALB	Homo sapiens	physical association	protein - protein
P02768	KRT13 interacts with ALB	Homo sapiens	physical association	protein - protein
P02768	CST3 interacts with ALB	Homo sapiens	physical association	protein - protein
P02768	ALB interacts with CRYAB	Homo sapiens	physical association	protein - protein
P02768	DCC interacts with ALB	Homo sapiens	physical association	protein - protein
P02768	KRT9 interacts with ALB	Homo sapiens	physical association	protein - protein
P02768	KRT6A interacts with ALB	Homo sapiens	physical association	protein - protein
P02768	CACNA1I interacts with ALB	Homo sapiens	physical association	protein - protein
P02768	SLC1A5 interacts with ALB	Homo sapiens	physical association	protein - protein
P02768	TSC22D1 interacts with ALB	Homo sapiens	physical association	protein - protein
P02768	CFB interacts with ALB	Homo sapiens	physical association	protein - protein
P02768	FGA interacts with ALB	Homo sapiens	physical association	protein - protein
P02768	FCGRT interacts with ALB	Homo sapiens	association	protein - protein
P09237	CD44 interacts with MMP7	Homo sapiens	physical association	protein - protein
P09237	MMP7 physically interacts with FASLG	Homo sapiens	physical interaction	protein - protein
P09237	ZBTB33 interacts with MMP7	Homo sapiens	physical association	protein - dna
P09237	HBEGF interacts with MMP7	Homo sapiens	physical association	protein - protein
P09237	Cleavage reaction involving DEFB4A and MMP7	Homo sapiens	cleavage reaction	protein - protein
P09237	Cleavage reaction involving MMP7 and Defb2	Homo sapiens/Mus musculus	cleavage reaction	protein - protein
P09237	Cleavage reaction involving DEFA1 and MMP7	Homo sapiens	cleavage reaction	protein - protein
P09237	Cleavage reaction involving DEFB1 and MMP7	Homo sapiens	cleavage reaction	protein - protein
P09237	Cleavage reaction involving MMP7 and Defb1	Homo sapiens/Mus musculus	cleavage reaction	protein - protein
P09237	MMP7 physically interacts with BCAN	Homo sapiens	physical interaction	protein - protein
P09237	MMP7 interacts with FASLG	Homo sapiens	physical association	protein - protein
P15086	CPB1 physically associates with CPB1	Homo sapiens	physical association	protein - protein

P21399	Colocalization of ACO1 and PSMG1	Homo sapiens	colocalization	protein - protein
P21399	APP physically interacts with ACO1	Homo sapiens	physical interaction	protein - protein
P21399	Genetic interaction between ACO1 and HDAC7	Homo sapiens	additive genetic interaction defined by inequality	dna - dna
P21399	Colocalization of STRAP and ACO1	Homo sapiens	colocalization	protein - protein
P21399	Colocalization of LGALS1 and ACO1	Homo sapiens	colocalization	protein - protein
P21399	Colocalization of DCPS and ACO1	Homo sapiens	colocalization	protein - protein
P21399	Colocalization of FUBP1 and ACO1	Homo sapiens	colocalization	protein - protein
P21399	Colocalization of ACO1 and C12orf57	Homo sapiens	colocalization	protein - protein
P21399	Colocalization of ACO1 and HIST1H2AE	Homo sapiens	colocalization	protein - protein
P21399	ACO1 interacts with AKT1	Homo sapiens	physical association	protein - protein
P21399	ACO1 interacts with POLR2F	Homo sapiens	transcriptional regulation	protein - dna
P21399	Colocalization of ACO1 and CTPS1	Homo sapiens	colocalization	protein - protein
P21399	ACO1 interacts with UBC	Homo sapiens	physical association	protein - protein
P21399	FBXL5 interacts with ACO1	Homo sapiens	physical association	protein - protein
